# Genome-based insights into metal co-resistance in Amazonian mercury-resistant bacteria: ecological lifestyle and biosafety implications for bioremediation

**DOI:** 10.1128/spectrum.03127-25

**Published:** 2026-04-30

**Authors:** Maria Camila Escobar, Juan Pablo Niño-Garcia, Alejandro Acosta-González, Carolina Díaz-Cárdenas, Yaneth Vasquez, Silvia Marques, Gladys Inés Cardona

**Affiliations:** 1 Laboratorio de Biotecnología y Recursos Genéticos, Instituto Amazónico de Investigaciones Científicas SINCHI 561453, Bogotá, Colombia; 2 Escuela de Microbiología, Universidad de Antioquia UdeA 525244 , Medellín, Colombia; 3 Facultad de Ingeniería, Universidad de La Sabana, Chía, Colombia; 4 Convergence Science and Technology Cluster, Universidad Central 27979, Bogotá, Colombia; 5 Department of Biotechnology and Environmental Protection, Estación Experimental del Zaidín, Consejo Superior de Investigaciones Científicas 73025, Granada, Spain; Gujarat Biotechnology University, Gandhinagar, Gujarat, India

**Keywords:** mercury bioremediation, *mer *operon, mobile genetic elements, metal-resistance genes, antibiotic resistance genes, co-selection, Amazonian environmental isolates, genomic biosafety

## Abstract

Mercury (Hg) pollution from artisanal gold mining represents a serious environmental and public health problem, especially in the Amazon Basin. Although mercury-resistant bacteria provide significant detoxification potential, their deployment needs genomic screening to assess both ecological lifestyle and biosafety. In this study, we analyzed the genomes of two highly mercury-resistant strains, *Pseudomonas paracarnis* TP30 and *Burkholderia contaminans* TR100, isolated from Hg-contaminated sediments in the Colombian Amazon. Both genomes contain broad-spectrum *mer* operons flanked by mobile genetic elements but differ in structure: TP30 encodes phage-associated mobility genes within a chromosomal *mer* locus, while TR100 has multiple metal resistance genes (MRGs) and a plasmid-borne *mer* operon embedded in a transposon. Despite the widespread presence of MRGs, both genomes show remarkably low levels of antibiotic resistance genes (ARGs), which contrasts with typical ARG–MRG co-selection patterns. Lifestyle prediction and gene-content analysis show that TP30 is a soil-adapted, environmentally persistent strain. At the same time, TR100, despite exhibiting a mixed ecological signal typical of the *Burkholderia cepacia* complex, lacks key virulence factors and displays features consistent with a non-pathogenic lifestyle. Our genomic analyses indicate that TP30 and TR100 harbor desirable traits for mercury bioremediation, including broad-spectrum Hg resistance, low antibiotic resistance gene content, and the absence of major pathogenicity markers, making them promising candidates for further evaluation under controlled conditions.

**IMPORTANCE:**

Mercury pollution from artisanal gold mining poses a significant threat to ecosystems and human health worldwide, particularly in vulnerable regions like the Amazon. Robust mercury-resistant bacteria offer a sustainable solution for detoxifying contaminated environments, but their potential application requires careful genomic assessment to minimize biosafety risks. This study integrates comparative genomics, resistance profiling, and lifestyle prediction to evaluate two highly mercury-resistant bacterial isolates from mining sediments. We show that these strains harbor broad-spectrum mercury resistance genes with low antibiotic resistance burdens and lack key virulence markers, supporting their suitability as environmentally adapted candidates. By coupling metal detoxification potential with genomic screening, our work highlights a framework for identifying promising, high-performance candidates for bioremediation, advancing microbiological solutions to address metal pollution in impacted ecosystems.

## INTRODUCTION

Mercury (Hg) pollution caused by artisanal and small-scale gold mining poses a serious threat to ecosystems and public health, particularly in the Amazon basin, where Hg is widely used in gold (Au) amalgamation (1). An estimated 2,000 tons of mercury are released annually at the global scale, with substantial fractions discharged directly into soils, rivers, and the atmosphere during Au–Hg amalgam heating (2). Once in the environment, Hg undergoes complex transformations largely mediated by microbial communities, including methylation to toxic methylmercury (MeHg), demethylation, and redox conversions (3). Among these, the microbial conversion of Hg(II) to volatile Hg(0) is of particular interest for remediation (4), as it reduces MeHg bioaccumulation risks (5) and its toxicity to humans and wildlife (6).

This detoxification capacity is encoded in the *mer* operon, a modular and variable genetic system found in many environmental bacteria (7). At its core, the *merA* gene encodes an NADPH-dependent mercuric reductase that converts Hg(II) to Hg(0). Broad-spectrum operons also include *merB*, which cleaves carbon–mercury bonds in organomercurial compounds like MeHg, releasing Hg(II) for subsequent reduction by MerA. Additional genes mediate periplasmic binding (*merP*), transport (*merT*/*C*/*F*/*E*), regulation (*merR* and *merD*), and resistance to hydrophobic organomercurials (*merG*) (7). The operon’s structure varies widely across taxa and can be located on chromosomes, plasmids, or mobile transposons (e.g., Tn21 and Tn501 of the Tn3 family [8]), which facilitates its horizontal gene transfer (HGT) and strains’ adaptation to contaminated environments. This plasticity and mobility make it a powerful model to study adaptation under metal stress and a promising tool for bioremediation (9).

The genomic plasticity of mercury-resistant bacteria enables adaptation to contaminated environments but also entails important implications for co-selection dynamics. Genes conferring resistance to mercury often co-occur with resistance genes to other metals (metal resistance genes [MRGs], e.g., to cobalt, zinc, cadmium, and copper), and antibiotics (antibiotic resistance genes [ARGs]), particularly when embedded within mobile genetic elements (MGEs, e.g., conjugative plasmids, transposons) (10). This co-localization may enable co-selection. Thus, selective pressure from one compound (e.g., Hg) promotes the persistence of unrelated resistance traits, including antibiotic resistance (11). Co-selection is especially concerning in bioremediation contexts, as the environmental release of resistant bacteria could inadvertently contribute to ARG dissemination (12). Studies have shown that over 70% of plasmids carrying mercury resistance genes also carry ARGs (13) and that *mer* operons can be transferred naturally in environmental settings (14). At the same time, co-occurrence with MRGs may confer a competitive advantage to these bacteria in multicontaminated environments, enhancing their bioremediation potential (15). Therefore, evaluating the balance between functional benefits and biosafety risks is essential when considering resistant strains in environmental applications (12).

Along with the changes in the physicochemical properties and microbial diversity of soils and sediments in affected Amazonian ecosystems (16), metagenomic studies have revealed an increasing abundance of MRGs and ARGs in Amazonian samples (17). These findings suggest an accelerated adaptation to stress and a competitive advantage for colonizing disturbed Amazonian environments, possibly mediated by the transfer of resistance genes through HGT (18). Notably, bacteria carrying the *mer* operon are not restricted to Hg-contaminated sites; they occur across diverse niches, from clinical settings and wastewater bioreactors to natural habitats, highlighting broad ecological versatility (19). This versatility makes them applicable models for examining how resistance traits align with different bacterial lifestyles.

Beyond the co-selection of antibiotic and metal resistance genes, anthropogenic environmental pressures can drive diverse bacterial lifestyles to converge toward more pathogenic modes (20). Recent comparative-genomics approaches that model presence–absence patterns of orthologous gene families now predict lifestyle categories with high accuracy, providing a first-pass assessment of whether a strain is more likely to behave as plant-associated, free-living, or human-opportunistic (21). Nevertheless, the genetic determinants that separate free-living from pathogenic lifestyles remain poorly resolved for many genera. *Burkholderia* illustrates this continuum: while members of the *Burkholderia cepacia* complex cause severe infections in patients with cystic fibrosis or those who are immunocompromised (22), closely related isolates inhabit soils and other niches, where they retain plant-beneficial traits (23–25). *Pseudomonas* is equally versatile, with over 300 described species that span serious human, animal, and plant pathogens (26–28), as well as classic plant-growth promoters (26). Isolates from both genera have even been deployed in metal bioremediation (29, 30), underscoring how a single taxon can range from a harmful pathogen to an environmentally beneficial organism.

To evaluate potential microbial candidates for mercury bioremediation in this context, we analyzed the genomes of two highly Hg-resistant strains, *Pseudomonas paracarnis* TP30 and *Burkholderia contaminans* TR100, which were previously isolated from sediments in two regions of the Colombian Amazon affected by AGM with different levels of Hg contamination (31). Both isolates exhibited high mercury tolerance, with minimal inhibitory concentrations (MICs) of 64 and 71 mg HgCl₂ L⁻¹, respectively. For comparison, published MIC ranges for environmental isolates are markedly lower: *Bacillus* spp., 6.25–27 mg HgCl₂ L⁻¹; *Serratia* spp., 12.5–50 mg HgCl₂ L⁻¹; and *Pseudomonas* spp., 25–100 mg HgCl₂ L⁻^1^ (32–35). These values place TP30 and TR100 at the upper end of reported mercury resistance. In addition, both strains showed progressive induction of *merA* expression in response to increasing mercury concentrations, resulting in significant mercury-reduction activity (31).

The main objectives of this study were to (i) characterize the genomes of two highly mercury-resistant bacterial strains, *P. paracarnis* TP30 and *B. contaminans* TR100, isolated from Hg-contaminated Amazonian sediments, and (ii) evaluate the genetic determinants underlying their Hg resistance mechanisms, co-resistance potential, and predicted ecological lifestyles to assess their suitability for mercury bioremediation. To achieve these aims, we combined comparative genomics with phylogenetic and lifestyle prediction approaches, contrasting TP30 and TR100 with 151 related strains from diverse environments.

## MATERIALS AND METHODS

### Morphology characterization

*Pseudomonas* sp. TP30 and *Burkholderia* sp. TR100 strains were isolated in a previous study (31) as Hg-resistant bacteria from superficial and interstitial sediment collected in Tarapacá and Taraira (in the Colombian Amazon region), respectively. These strains are preserved in the COLMIS microorganism collection at the Amazonian Scientific Research Institute SINCHI (Collection Single Registry number 282 of 2022). Additionally, the COLMIS collection is protected under a contract for access to genetic resources and derived products for commercial purposes (number 277 of 2019, Ministry of Environment and Development of Colombia).

Scanning electron microscopy (SEM) and transmission electron microscopy (TEM) with energy-dispersive X-ray spectroscopy (EDS) analyses were carried out by the Laboratory for Transmission Electron Microscopy Equipment (Universidad de Antioquia, Colombia). TP30 and TR100 strains were reactivated in Luria–Bertani broth (bacteriological tryptone 10 g L^−1^, yeast extract 5 g L^−1^, and NaCl 10 g L^−1^) containing Hg (40 mg HgCl_2_ L^−1^) and incubated for 48 h at 30°C with agitation (100 rpm). A culture medium without mercury was used as a control. Afterward, the cells were collected by centrifugation (1,000 rpm, 5 min, 4°C) and washed three times with sterile water. Pelletized cells were fixed in 2.5% (vol/vol) glutaraldehyde and then visualized by SEM and TEM-EDS, as previously described (36) (Fig. S1).

### DNA extraction and whole-genome sequencing

High-molecular-weight (HMW) DNA from the two bacterial strains was extracted using the Quick-DNA HMW MagBead kit (ZYMO Research, USA), following the manufacturer’s instructions, which included resuspending the bacterial cells in DNA/RNA Shield (ZYMO Research). HMW DNA was evaluated on the 4150 TapeStation System using genomic DNA ScreenTape and reagents (Agilent, Santa Clara, CA, USA).

Genomic DNA was sequenced using two complementary technologies: Nanopore and Illumina sequencing. First, in-house Nanopore whole-genome sequencing was used to obtain long sequences (10–100 kb for long-read sequencing mode and 100–300 kb for ultra-long-read sequencing). Nanopore libraries were generated using the Native Barcoding Expansion Kit (EXP-NBD104) and the Ligation Sequencing Kit (SQK-LSK109) without size selection (Oxford Nanopore Technologies, UK). Pooled libraries were quantified and quality-assessed using a Qubit 3.0 fluorometer (Invitrogen, USA) and an Agilent 4150 TapeStation (Agilent Technologies, Massachusetts, USA), respectively, before sequencing. Final libraries were loaded onto an R9.4.1 flow cell (FLO-MIN106D), and whole-genome sequencing was performed for 48–72 h on a MinION Mk 1B device (Oxford Nanopore Technologies). Base calling of raw Nanopore signals was performed onboard using Guppy (v5.0.11) in high-accuracy mode, the standard configuration available in MinKNOW during the sequencing runs. Reads with a minimum PHRED quality score of ≥7 were retained for downstream analysis (>95%) (Fig. S2A and B). Mean read length was recorded for quality-control purposes—8.5 and 4.8 kb for TP30 and TR100, respectively—and used to verify library integrity before downstream analyses (Fig. S2C and D). The quality of the sequences obtained with ONT was evaluated with pycoQC (37) and NanoPlot (v1.18.2) by barcode.

Second, the Illumina NovaSeq PE150 sequencing was performed at Novogene (USA) to obtain high-quality short sequences (350 bp). For Illumina quality control, we utilized paired reads free of adapter contamination and uncertain nucleotides (with more than 10% N in either read), along with high-quality nucleotides (base quality above 5). Following this process, 83.95% (18,812,436 bp) and 99.8% (13,413,394 bp) of the sequences from the TP30 and TR100 strains, respectively, remained.

### Sequence assembly, annotation, and analysis

After verifying the quality of ONT sequences, NECAT (38) was used as an error corrector for the long reads. Different assembly tools were utilized in the assembly process: Flye 2.9.1-b1780 (39), NECAT (38), CANU 2.1.1 (40), and NGSEP 4.2.1 (41). The quality of the assemblies was evaluated using QUAST (v5.2.0) (42), and completeness was assessed with BUSCO 5.4.4 (43). The best genome assemblies were polished with Illumina sequences using the GenomeIndexer, ReadsAligner, SingleSampleVariantsDetector, and IndividualGenomeBuilder commands of NGSEP (v4.2.1) (41), along with SortSam from Picard tools (v1.41). Assembled genomes were reevaluated for quality and completeness using QUAST and BUSCO, respectively (Fig. S3A and B; Table S1). Genome sequences are available in the NCBI GenBank with the BioProject number PRJNA1445321, and the accession numbers SAMN56786084 and SAMN56786085 for *P. paracarnis* TP30 and *B. contaminans* TR100, respectively.

The resulting complete genome sequences were annotated using Rapid Annotation using Subsystem Technology (RAST) (44) (Fig. S4; Table S2) and PROKKA (v1.11) (45), but the ARG search was conducted with the CARD database (46) and DeepARG (47). Circular visualization of the genomes was performed with Genovi 0.4.3 (Fig. S3C and D) (48), and selected genes were mapped using Proksee (Tables S3 and S4) (49).

### Multilocus phylogenetic analysis

For the multilocus phylogenetic analysis (MLSA) and following the strategy of Glaeser and Kämpfer (50), 10 essential genes from the genomes of strains TP30 and TR100 were selected. The selected genes were *gyr*A, *gyr*B, *rec*A, *rpo*A, *rpo*B, *atp*A, *dna*K, *fus*A, *lep*A, and *pyr*G. The genes of strains TP30 and TR100 were extracted from the RAST annotation. In contrast, the genes of the closely related strains, selected based on ANI and Tetra classification analyses performed with the JSpecies program (51), were obtained from the NCBI genome annotations. For the construction of the trees, the sequences were aligned using the WebPRANK program (52), and the visualization of the trees was performed with the IcyTree program (53), employing the default parameters.

### Genomic and *mer* operon comparison analysis

To explore genomic plasticity and co-resistance potential in mercury-resistant bacteria, we performed a comparative analysis of shared and accessory gene content using PPanGGOLiN 2.1.1 (54) and the *mer* operon. A total of 71 genomes from *Pseudomonas* spp. were analyzed, including 30 genomes of *P. paracarnis* (such as *P. paracarnis* TP30 and 15 unclassified or misclassified strains), 16 of *P. lactis* (including one unclassified strain), and 23 of *P. carnis* (including six unclassified or misclassified strains). Also, a total of 80 genomes of *B. contaminans* strains (including TR100) were used. Genome selection was based on ANIb values ≥95% as performed in the JSpecies program (51). It included outgroup strains with lower ANIb values to improve tree rooting (*P. paralactis* SWRI70, *P. paralactis* DSM 29164, *B. contaminans* AuBur16, and *B. contaminans* GalA64; see Supplementary Excel Tables S5 and S6). Selected strains were isolated from diverse environments (Tables S7 and S8).

All genomes were annotated using RAST (44), and ARGs were predicted with DeepARG (47). We also identified MRGs (e.g., mercury, copper, and cobalt–zinc–cadmium) and MGEs such as phage genes (e.g., phage tails, capsid proteins, and phage packing machinery), insertion/transposon genes (e.g., transposases and recombinase/integrase), and plasmid-related genes (plasmid-related proteins, conjugative transfer endonucleases IncQ, replication initiator, and stabilization proteins).

The gene presence–absence matrices derived from the PPanGGOLiN analysis were used to calculate Bray–Curtis dissimilarity matrices using the proxy package (v0.4.27). Hierarchical clustering and circular cladograms were generated in R (v4.4.1) (55) using the stats package and RStudio (v2024.9.0.375) (56).

To test whether genomic composition was structured by isolation source or by the presence of the *mer* operon, PERMANOVA analyses were performed using the adonis2 function in the vegan package (v2.6.8), based on Bray–Curtis distance matrices and 10,000 permutations. Homogeneity of multivariate dispersion was assessed using the betadisper function (vegan) to verify the robustness of PERMANOVA results. Significant PERMANOVA results were followed by Bonferroni-corrected Dunn’s post hoc tests (dunnTest) to evaluate pairwise differences.

The circular heatmap displaying the number of ARGs, MRGs, and MGE-associated genes per genome was created using the ComplexHeatmap package (v2.20.0) (57). For visualization, gene counts were standardized per genome using *z*-scores (clipped to ±4) and displayed as concentric heat rings. Principal component analysis (PCA) was applied to scaled gene-category abundance data to explore multivariate patterns associated with isolation source and *mer* operon presence. PCA was performed using standard eigenvalue decomposition as implemented in FactoMineR, and results were visualized using biplots.

Differences in gene counts (ARGs, MRGs, and mobile genetic elements) among species, isolation sources, and *mer* operon presence were assessed using non-parametric Kruskal–Wallis tests, given the discrete nature and non-normal distribution of count data. When global tests were significant, pairwise comparisons were performed using Dunn’s post hoc test with Bonferroni correction. All analyses were conducted in R.

Additional exploratory analyses were conducted in R, including Pearson’s (*r*) and Spearman’s (*ρ*) correlations (Hmisc v5.1.3 [58]) to evaluate associations between resistance genes and mobile genetic elements (only associations significant under both metrics are reported), and non-metric multidimensional scaling (metaMDS; vegan v2.6.8 [59]) to explore global abundance patterns.

*mer* operons were identified in 30 and 48 genomes of *Pseudomonas* spp. and *B. contaminans*, respectively, by screening the annotated gene neighborhoods for *merA* and then examining upstream and downstream regions (~10 kb) for the presence of accessory genes (*merB*, *merR*, *merT*, *merP*, *merC*, *merD*, *merE*, and *merG*) and mobile elements.

Operon boundaries were defined based on synteny and functional annotation. The structural organization of operons was visualized using the gggenes package in ggplot2 (60). For each operon, we annotated flanking mobile elements (e.g., *tniABQ*, transposases, and integrases) and co-occurring MRGs (e.g., cobalt/zinc/cadmium [*czc*], *cop*, and *chr*). Genomic neighborhoods were plotted to assess operon conservation, rearrangements, and potential HGT signatures.

Additionally, consensus amino acid sequences of shared operon-encoded proteins (MerR, MerT, MerP, and MerA) were used to make a phylogenetic analysis using Geneious Prime 2024.0.7. Phylogenetic trees were constructed using the neighbor-joining method implemented in Geneious Prime, based on MUSCLE protein alignments of Mer proteins (MerR, MerT, MerP, and MerA). Pairwise distances were calculated using the distance correction implemented in Geneious for protein alignments (Jukes–Cantor). Node support was assessed using non-parametric bootstrap resampling (10,000 replicates), and bootstrap values of >50% are shown at the nodes. The consensus *mer* operon proteins of *Aquipseudomonas alcaligenes* GD03990 38 and *Caballeronia mineralivorans* PML1(12) were employed as outgroups for *Pseudomona*s spp. and *B. contaminans*, respectively.

### Lifestyle prediction and identification of key signature genes

To infer the ecological lifestyles of our strains, we independently ran bacLIFE (21) on the two genus-specific data sets (*Pseudomonas* spp. and *B. contaminans*) comprising 151 assemblies, which were processed within the bundled Conda + Snakemake environment. For each genus, genomes were first annotated using Prokka v1.14.6 (default parameters). The predicted proteins were then de-replicated with MMseqs2 linclust (90% identity, 80% coverage) and clustered into orthologous gene families using MCL (inflation = 3.0), yielding 5,755 families for the *Pseudomonas* data set and 6,399 for *B. contaminans*. Lifestyle prediction was performed using bacLIFE’s pre-trained random-forest classifiers. Model performance was evaluated within the bacLIFE framework using fivefold cross-validation and assessed through the receiver operating characteristic (ROC) curves, reporting the mean area under the curve (AUC ± SD), where AUC = 0.5 indicates random classification. The statistical significance of lifestyle-associated structuring in gene content was assessed using PERMANOVA (adonis2, vegan) under a reduced model with free permutations (999 permutations), as implemented within the bacLIFE analytical framework.

Isolation-source metadata provided five lifestyle labels (human-opportunistic, plant-associated, food-associated, insect-associated, and soil/water free-living), which were used by bacLIFE’s pre-trained random-forest models to return posterior probabilities for each lifestyle and a ranked list of lifestyle-associated genes (LAGs) based on SHAP values. Genes present in ≥60% of genomes within a lifestyle were designated restrictive LAGs, whereas those present in 40%–59% were designated lax LAGs; genes meeting a threshold in multiple lifestyles were counted as shared.

To visualize genome-wide gene-content relationships underlying lifestyle predictions, the genus-specific presence–absence matrices used by bacLIFE were converted to Bray–Curtis dissimilarity matrices and ordinated using principal coordinate analysis in R (vegan).

## RESULTS AND DISCUSSION

### Morphological and genomic characterization of TP30 and TR100

*Pseudomonas paracarnis* TP30 and *Burkholderia contaminans* TR100 were previously isolated as Hg-resistant bacteria from Amazonian sediments in Tarapacá and Taraira, Colombia (31). TP30 exhibited elongated rod-shaped cells, whereas TR100 showed shorter, plumper morphologies with abundant cytoplasmic inclusion bodies consistent with polyhydroxyalkanoates (Fig. S1). No intracellular mercury accumulation was detected in either strain, suggesting that mercury resistance relied on alternative mechanisms such as enzymatic Hg volatilization.

**Fig 1:**
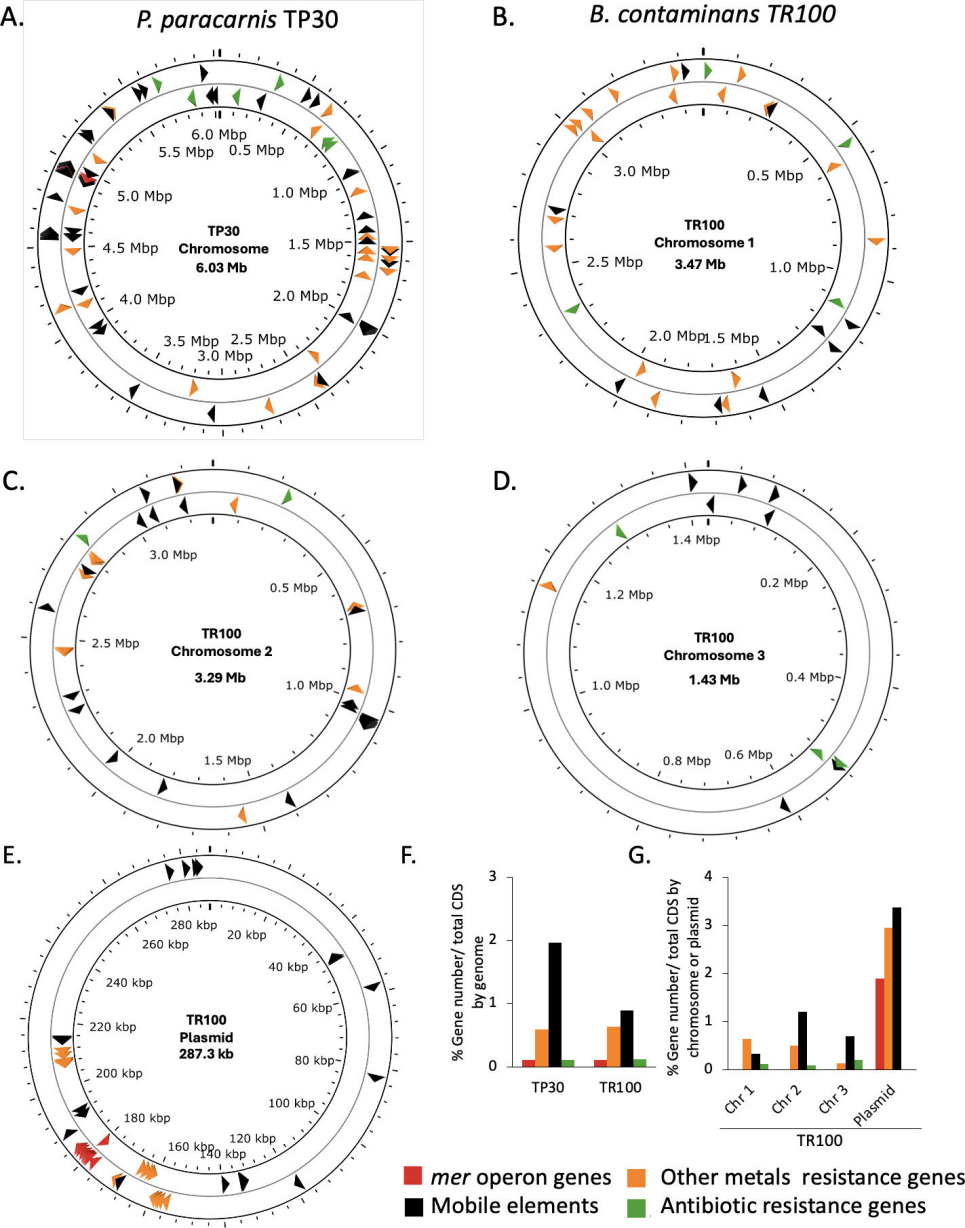
Distribution and mapping of genes related to toxic compound resistance and mobile elements in two Amazonian isolates. (**A**) *Pseudomonas paracarnis* TP30 (chromosome). (**B–E**) *Burkholderia contaminans* TR100: (**B**) chromosome 1, (**C**) chromosome 2, (**D**) chromosome 3, and (**E**) plasmid. Colored arrows mark annotated loci by category: *mer* operon genes (red), other metal resistance genes (orange), antibiotic resistance genes (green), and mobile genetic elements (black). Tick marks indicate genome coordinates. (**F**) Relative proportion of each category as a percentage of total CDSs in TP30 and TR100. (**G**) Same metric for TR100 partitioned by replicon (chromosomes 1–3 and plasmid).

Nanopore sequencing followed by Illumina polishing yielded complete, high-quality assemblies: a single circular 6.0 Mb chromosome for TP30 and a multipartite 8.5 Mb genome for TR100 comprising three chromosomes and one putative plasmid (Fig. 1; Fig. S3). This genomic architecture is consistent with the known multichromosomal organization of *Burkholderia* species (61). Assemblies generated with CANU and FLYE were retained for downstream analyses based on their high BUSCO completeness scores (Fig. S3A). Taxonomic assignments were confirmed by ANI (>95%) and MLSA (Fig. S5; Tables S5 and S6).

Functional annotation using RAST and Prokka revealed broadly similar functional profiles dominated by transcription, amino acid metabolism, and cell envelope processes. Notably, the TR100 plasmid was enriched in recombination, mobilome, and inorganic-ion transport genes (Fig. S4), suggesting a role in adaptive functions. Together, these high-quality assemblies and annotations provide a robust genomic framework for the comparative, co-selection, and lifestyle analyses presented in the following sections.

### Distribution of resistance genes and MGEs

It is widely accepted that resistance to mercury, other metals, and antibiotics is often associated through a process called co-selection (e.g., co-resistance *sensu* [11]). This process depends on the presence and clustered distribution of genes associated with MGEs (62). We tested to what extent the genes related to these functions were spatially clustered in the replicons of TP30 and TR100.

In general, we found that the metal and antibiotic resistance genes were scattered across the TP30 genome and between the TR100 chromosomes (Fig. 1A through E). However, clear differences emerged in the local organization of resistance determinants. In TP30, antibiotic resistance genes (including aminoglycoside, beta-lactam, phosphomycin, and fluoroquinolone resistance) showed localized clustering on the chromosome (Fig. 1A; Table S3), whereas metal resistance genes, particularly those conferring resistance to copper, silver, and cobalt/zinc/cadmium, were preferentially clustered near the *mer* operon on the TR100 plasmid (Fig. 1E; Table S4). A similar but more limited co-localization pattern was observed in TP30, restricted mainly to copper and chromate resistance genes.

The distribution of MGEs further highlighted contrasting genomic strategies between strains. TP30 contained approximately twice the relative proportion of MGEs across its chromosome compared to TR100 (Fig. 1F). In contrast, the TR100 plasmid was markedly enriched in MGEs, showing 10-, 3-, and 5-fold higher proportions than chromosomes 1, 2, and 3, respectively (Fig. 1G). Notably, *mer* operons were consistently flanked by MGEs in both strains, regardless of their chromosomal or plasmid location (Fig. 1A and E), supporting their mobilizable nature.

These patterns partially support co-resistance hypothesis between mercury and other resistance traits, particularly in TR100, where the plasmid-borne clustering of metal resistance genes and MGEs suggests a higher potential for co-selection. Similar configurations have been reported in other *B. contaminans* strains harboring multiple plasmids (63, 64). In bacteria with multipartite genomes (estimated to represent ~10% of bacterial species [65, 66]), core functions are typically encoded on the primary chromosome, while secondary replicons contribute adaptive flexibility under fluctuating environmental conditions (67). In TR100, this organization may explain the elevated abundance of MGEs on chromosome 2 (Fig. 1G) and the localization of the *mer* operon on a plasmid, which likely has a higher copy number than chromosomal loci and may contribute to the higher mercury resistance observed in this strain (31).

At the same time, plasmid-borne resistance determinants may be more prone to loss in the absence of selective pressure (68), highlighting a potential trade-off between resistance strength and long-term stability. In contrast, the predominantly chromosomal and dispersed distribution of resistance genes in TP30 suggests greater inheritance stability and reduced risk of segregational loss (69). Although minimal selective concentrations were not experimentally determined here, sub-inhibitory levels of metals and antibiotics are known to maintain resistance determinants in environmental bacteria (69, 70).

From an applied perspective, these contrasting genomic architectures have important implications for bioremediation. Chromosomally encoded resistance in TP30 may confer greater long-term stability in remediation settings, whereas plasmid-associated resistance in TR100 may provide higher resistance levels but at the cost of potential instability when selective pressures decline. Together, these results reveal distinct organizational strategies for resistance genes and MGEs in TP30 and TR100, dispersed and chromosomal versus clustered and plasmid centered, which shape their co-resistance potential, stability, and suitability for bioremediation applications. Experimental validation of these predictions, including stability and performance assays under controlled conditions, would be required to confirm their functional implications.

### Structure, phylogeny, and genomic context of *mer* operons

To explore the mercury resistance determinants of TP30 and TR100 in the context of their closest relatives (ANIb >95%), we assembled two genus-specific genomic data sets comprising 71 *Pseudomonas* spp. and 80 *B. contaminans* genomes (see details in the methods section). These data sets served as the foundation for a series of comparative analyses aimed at determining the phylogeny of Mer proteins, genome diversity, co-selection patterns, and lifestyle predictors.

Complete *mer* operons were detected in 42% of *Pseudomonas* spp. genomes and 60% of the *B. contaminans* genomes. Most operons displayed the canonical architecture, including the transcriptional regulator (*merR*), transport genes (*merT*, *merP*, *merC,* or *merF*), and the reductase (*merA*) (71). Several genomes carried multiple *mer* operons with distinct phylogenetic affiliations, consistent with acquisition through independent HGT events rather than intralineage duplication, a pattern also reported for the heavy metal-resistant strain *Cupriavidus metallidurans* CH34 (72). In this context, TP30 harbored exclusively chromosomal *mer* loci, whereas TR100 displayed a plasmid-associated configuration, highlighting distinct organizational strategies between the two isolates.

**Fig 2:**
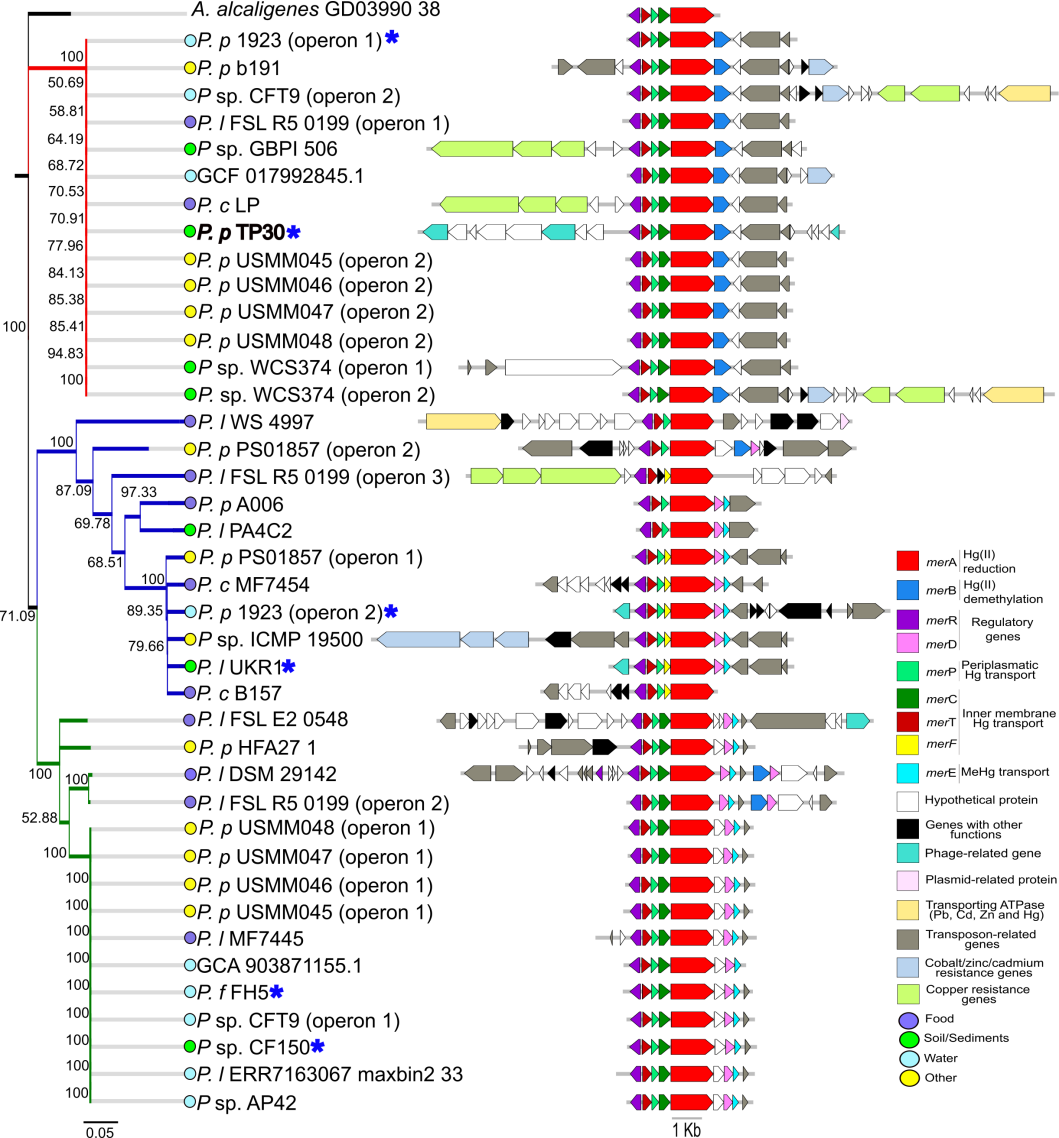
Phylogeny and synteny of complete *mer* operons in *Pseudomonas* spp. strains. (Left) Neighbor-joining consensus tree inferred from concatenated Mer proteins (MerR, MerT, MerP, and MerA); bootstrap values (>50%) are shown at the nodes. (Right) Linear diagrams of each operon, colored by gene function (legend, bottom right). Colored dots beside strain names indicate the reported isolation niche (food, soil/sediment, water, others). Scale bar, 0.05 substitutions per site; operon scale, 1 kb. The blue asterisks indicate that the strains were isolated from sites contaminated with Hg or other metals. For clarity, only genes associated with Hg resistance, co-resistance, or mobility are displayed; unrelated genes in the surrounding genomic region were excluded from the figure. The three cluster branches are distinctly colored as mentioned in the text.

Across *Pseudomonas* spp., *mer* operon synteny was largely congruent with Mer-protein phylogeny, suggesting dissemination of a relatively conserved *mer* module via horizontal transfer (Fig. 2). The TP30 operon clustered with 13 closely related strains sharing the *merR/TPCAB* architecture; in all members of this cluster, the operon was followed by a hypothetical protein and two transposon-related genes (red cluster, Fig. 2). Notably, TP30 was unique in being flanked on both sides by phage-derived genes, indicating insertion of the *mer* cluster within a remnant prophage. Additional *mer* operon configurations were observed: one cluster lacked *merC* but carried *merF* as a functional substitute when co-occurring with *merT* and *merP* (71) (the 11-strain blue cluster), while another included *merC*, *merD*, and *merE* but lacked *merB*, a configuration associated with resistance to inorganic rather than organomercurial mercury (green cluster, Fig. 2).

**Fig 3:**
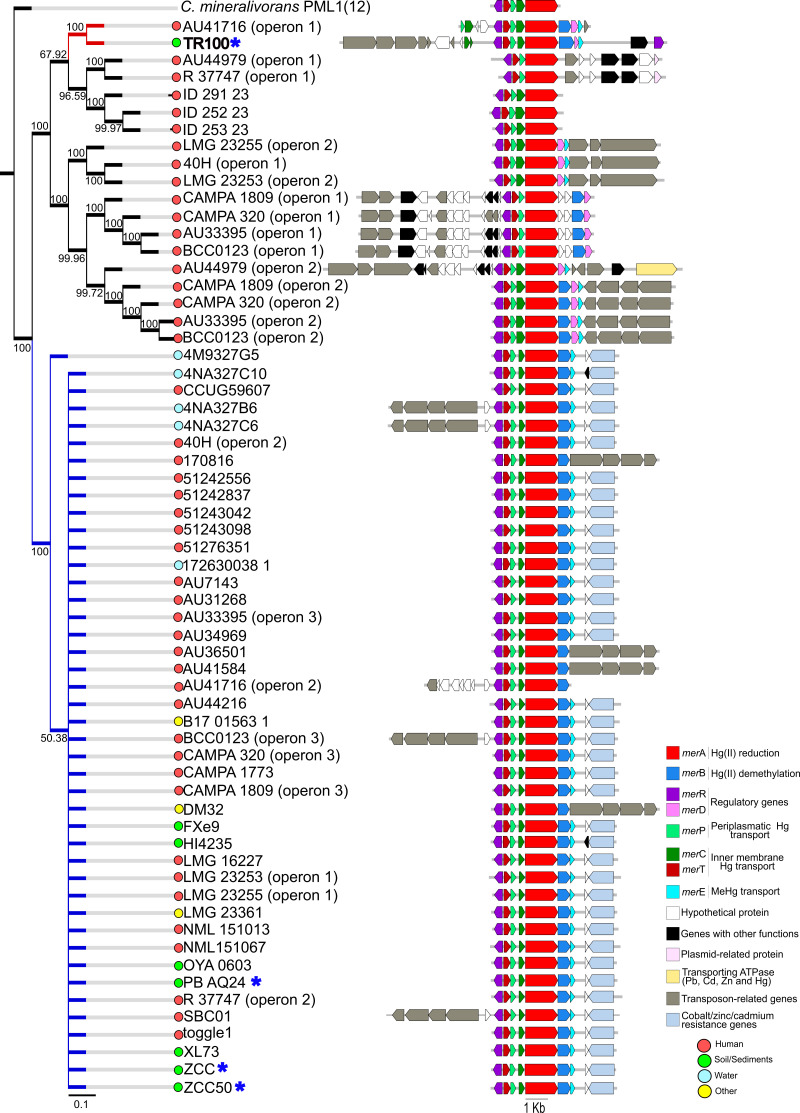
Phylogeny and synteny of complete *mer* operons in *B. contaminans* strains. (Left) Neighbor-joining consensus tree inferred from concatenated Mer proteins (MerR, MerT, MerP, and MerA); bootstrap values (>50%) are shown at the nodes. (Right) Linear diagrams of each operon, colored by gene function (legend, bottom right). Colored dots beside strain names indicate the reported isolation niche (human, soil/sediment, water, others). Scale bar, 0.05 substitutions per site; operon scale, 1 kb. The blue asterisks in the figure indicate that the strains were isolated from sites contaminated with Hg or other metals. For clarity, only genes associated with Hg resistance, co-resistance, or mobility are displayed; unrelated genes in the surrounding genomic region were excluded from the figure. The different cluster branches are distinctly colored, as mentioned in the text.

In *B. contaminans*, the TR100 *mer* operon encoded *merD* and *merE*, which are responsible for a co-repressor of operon expression and a transporter for Hg(II)/MeHg, respectively (7), and clustered with a divergent group closely related to operon 1 of strain AU41716 (red cluster, Fig. 3). Upstream of *merR*, TR100 contained the *tniA*/*B*/*Q* transposition module of Tn5053, a mobile element known to carry *mer* operons (71), indicating that its *mer* locus resides on a transposon embedded within a plasmid (Fig. 1E). Approximately 70% of *B. contaminans* operons grouped into a single cluster with conserved *merR*/*TPCABE* synteny, frequently followed by a *czc* resistance locus (blue cluster; Fig. 3). The *czc* locus encodes the *CzcCBA* efflux pump, which mediates divalent metal extrusion and has been linked to antibiotic cross-resistance via membrane permeability regulation (73). Its physical linkage with *mer* loci exemplifies co-resistance and may facilitate co-transfer and persistence of metal and antimicrobial resistance determinants under metal-rich conditions (74).

Both strains presented broad-spectrum *mer* operons, with the typical *merR*/*TPCAB(DE*) organization, conferring resistance to both inorganic and organic mercury compounds (7). As expected, neither operon contained *merG*, a gene associated with recently evolved operons and rarely detected in mesophilic bacteria (71). In contrast, the organomercurial lyase *merB* exhibited a strongly uneven distribution, being present in 69% of *B. contaminans* genomes but only 20% of *Pseudomonas* spp. genomes (Fig. 2 and 3; Tables S7 and S8). This pattern was independent of isolation source (Table S8) and is best explained by lineage-specific acquisition and retention of broad-spectrum *mer* operons in *B. contaminans* (7, 19). Consistent with prior work, *merB* was much less prevalent than *merA*, likely reflecting its relatively recent incorporation into mesophilic *mer* operons and its low metagenomic prevalence, comparable to that of Hg methylating microorganisms carrying the *hgcAB* cluster (75), which encodes two essential proteins for microbial mercury methylation: a corrinoid protein HgcA and an associated ferredoxin HgcB, together catalyzing the transfer of a methyl group to Hg(II) under anoxic conditions (76, 77).

Across both taxa, Mer-protein phylogenies grouped operons into clusters sharing conserved architectures and recurrent flanking genes, including transposases, plasmid- or phage-associated elements, and additional metal resistance determinants (Fig. 2 and 3). Notably, neither *mer* operon organization nor its genomic context was associated with the isolation source. However, clear taxon-specific differences emerged: additional metal resistance genes (e.g., copper and *czc* loci) were frequently found adjacent to *mer* operons in *B. contaminans* (≈61%) but were much less common in *Pseudomonas* spp. (≈20%). While *Pseudomonas* operons were more frequently flanked by diverse mobile genetic elements (Fig. 2), *B. contaminans* genomes showed stronger signatures of metal co-resistance and carried a higher average number of antibiotic resistance genes, suggesting a greater potential for co-selection and HGT in this lineage (Fig. 3) (78).

Together, these analyses show that *mer* operon architecture and genomic context are shaped primarily by lineage-specific evolutionary histories and mobile genetic elements rather than by isolation source. Distinct organizational strategies in *Pseudomonas* and *B. contaminans* highlight taxon-specific pathways for mercury resistance dissemination and co-resistance potential, providing a mechanistic foundation for the co-selection patterns examined in subsequent analyses.

### Comparative genomics across *Pseudomonas* and *Burkholderia* data sets

**Fig 4:**
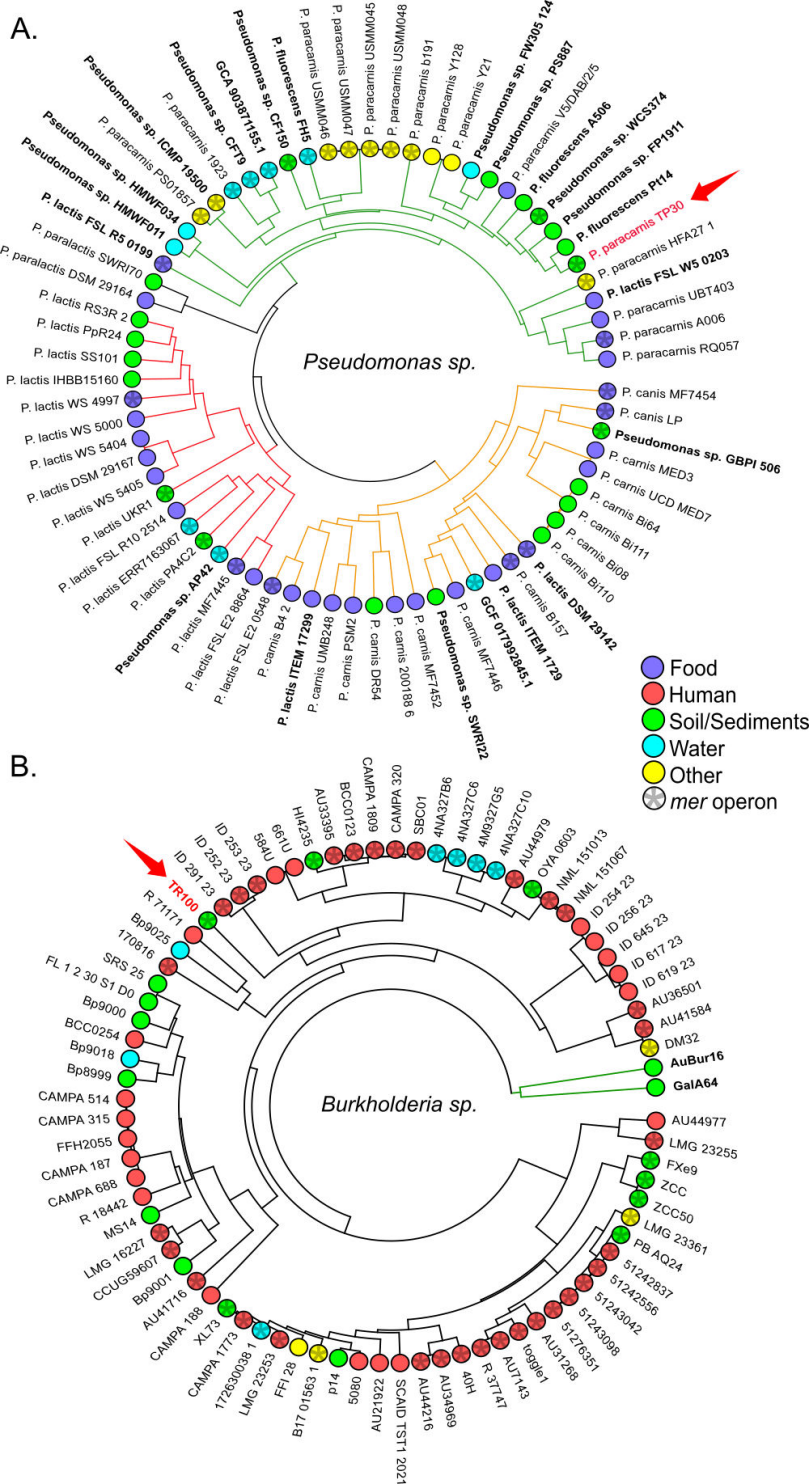
Cladograms based on Bray–Curtis dissimilarity distances using all genes of the genomic comparative analysis of the selected strains: (**A**) *P. paracarnis*, *P. carnis*, and *P. lactis*, and (**B**) *B. contaminans*. The cladograms specify isolation source (color code) and the presence of the *mer* operon (asterisk-filled dot). Three clades were clearly formed in the *Pseudomonas* spp. cladogram, corresponding to each of the selected species: *P. paracarnis*, *P. lactis*, and *P. canis*. Although some of the strains (indicated in bold) were originally misclassified (Tables S5 and S6), they appeared in the clades of the species to which they truly belonged.

So far, we have shown some evidence of co-resistance within the *mer* operons, with slight differences between strains (Fig. 2 and 3). To place these findings in a broader genomic context, we analyzed the complete genomes of the previously selected 71 *Pseudomonas* spp. and 80 *B. contaminans* genomes (Fig. 4; Tables S7 and S8). Comparative genomics revealed large, open pangenomes in both taxa (≈20% core genome), consistent with the metabolic versatility and niche breadth typical of environmental generalists (79, 80).

Hierarchical clustering based on Bray–Curtis distances showed no significant structuring by isolation source or *mer* operon presence in either genus (PERMANOVA, all *P* > 0.29), and this pattern was not driven by differences in within-group dispersion (betadisper, all *P* > 0.1; Fig. 4). Sampling was uneven across sources: in our data set, *B. contaminans* genomes were predominantly human associated (65%), whereas *Pseudomonas* spp. genomes were mostly food associated (42%). Given this imbalance, the absence of environment-driven clustering should be interpreted cautiously. Taken together with the frequent adjacency of *mer* loci to transposition/integron genes (Fig. 2 and 3), the observed patterns may be influenced by processes such as HGT mediated by mobile genetic elements (13, 81), but direct tests of mechanism are beyond the scope of these analyses.

Nonetheless, complete *mer* operons were detected in 42% of *Pseudomonas* spp. and 60% of *B. contaminans* genomes, predominantly from human- and food-associated sources (Fig. 4; Tables S7 and S8). This distribution indicates that mercury resistance is not restricted to contaminated environments, such as mining soils (31), but is also maintained in clinical and agri-food settings, likely through co-selection with antibiotic resistance and exposure to biocides and trace metals (82–85).

At a genome-wide scale, neither environmental origin nor the presence of the *mer* operon structured overall genomic composition; instead, mercury resistance appears embedded within open pangenomes and shaped primarily by HGT across ecological contexts. However, given that environmental pressures can act more strongly on specific resistance and mobility determinants, we next examined how co-selection signatures (MRGs, ARGs, and MGEs) varied across different ecological contexts.

### Environmental shaping of resistance, mobility, and ecological lifestyle signatures

**Fig 5:**
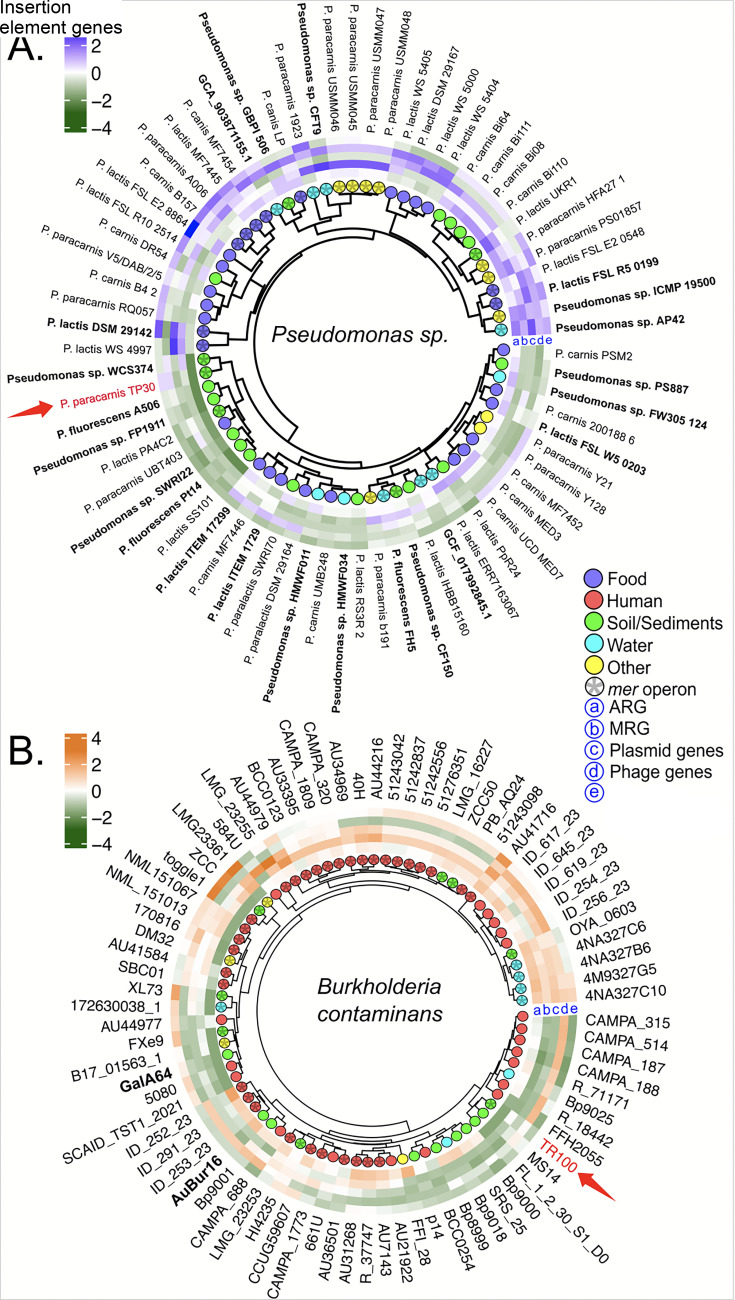
Gene-content clustering with standardized abundance of resistance and mobility markers. (**A**) Selected *P. paracarnis, P. carnis*, and *P. lactis*, and (B) selected *B. contaminans* genomes. Circular dendrograms are based on Bray–Curtis dissimilarity distances computed from gene-content profiles. Heat rings display standardized per-genome counts (*z*-scores, clipped to ±4) for each category: ARGs (a), MRGs (b), plasmid-associated markers (c), phage-associated markers (d), and insertion sequence genes (e). Color scales: blue (+4) to green (–4) in panel A; orange (+4) to green (–4) in panel **B**. Outer dots indicate isolation source, and an asterisk inside the dot marks genomes carrying a *mer* operon.

When focusing specifically on genes involved in co-selection, we identified 8,640 MRGs, 2,228 ARGs, and 13,433 MGEs. In *Pseudomonas* spp., PERMANOVA based on Bray–Curtis dissimilarities of gene-content profiles revealed a significant effect of isolation source on multivariate gene composition (*P* = 0.008; Fig. 5A; Fig. S6A). Bonferroni *post hoc* comparisons indicated that soil/sediment isolates differed significantly from other sources. Homogeneity of multivariate dispersion was confirmed for this comparison (betadisper, *P* > 0.6), supporting that the observed PERMANOVA signal reflects differences in gene-content composition rather than differences in within-group variability.

Despite high data dispersion, soil/sediment isolates exhibited the lowest mean gene counts per genome (Fig. 5A; Fig. S7B). High dispersion in gene counts among isolates likely reflects niche-specific selective pressures, consistent with evidence that microhabitat heterogeneity and local environmental stressors drive genomic variability and adaptive traits in *Pseudomonas* populations inhabiting contaminated soils and sediments (86). Moreover, this pattern aligns with reports that some metal-rich soils harbor streamlined resistance repertoires dominated by MRGs without concomitant ARGs enrichment, likely reflecting limited antibiotic/biocide inputs and mobilome composition (10).

Gene abundance patterns varied significantly (*P* ≤ 0.001), with *Pseudomonas* spp. displaying higher phage gene counts and *B. contaminans* showing increased numbers of insertion sequences (Fig. S7A). PCA of standardized gene abundances revealed a clear separation between *mer*-positive and *mer*-negative genomes in both taxa, supported by PERMANOVA (*Pseudomonas* spp.: *P* = 0.001, *R*² = 0.20; *B. contaminans*: *P* < 0.001, *R*² = 0.29; Fig. S6B and D). In *Pseudomonas* spp., this separation reflected shifts in gene-content composition rather than dispersion effects (betadisper, *P* > 0.3), whereas in *B. contaminan*s*,* it was accompanied by increased within-group heterogeneity among *mer-*positive genomes (betadisper, *P* = 0.013), consistent with enhanced genome plasticity.

Notably, *mer*-positive *Pseudomonas* strains exhibited markedly higher phage gene counts (83 ± 29.6), concentrated in the upper cladogram region (Fig. 5A; Fig. S7D). Similarly, *mer*-positive *B. contaminans* strains displayed elevated phage and insertion sequence counts (44.6 ± 14.4) (Fig. 5B; Fig. S7E). These taxon-specific patterns were supported by correlation analyses, which showed that in *Pseudomonas* spp., MRG abundance correlated positively with phage-associated genes (*ρ* = 0.45, *r* = 0.42, *P* < 0.001), whereas in *B. contaminans*, it correlated strongly with plasmids (*ρ* = 0.66, *r* = 0.62, *P* < 0.001) and insertion sequences (*ρ* = 0.61, *r* = 0.57, *P* < 0.001) (Fig. S8A, C, S9A and B). These results suggest that phage-mediated mobilization may be a key driver of gene flow in *Pseudomonas* spp., consistent with recent evidence showing widespread prophage carriage and functional contributions of phage-encoded genes in sediment-associated *Pseudomonas* populations, including deep-sea microbiomes (87). In contrast, the insertion sequence proliferation observed in *B. contaminans* likely reflects a plasmid-associated mobilization strategy, consistent with evidence that insertion sequence elements promote plasmid rearrangements and genome plasticity, thereby facilitating niche-specific adaptation (88, 89). Co-selection evidence between MRGs and ARGs was restricted to *Pseudomonas* spp., albeit weak (*ρ* = 0.28, *r* =0.27, *P* <0.05; Fig. S8D). While correlations do not confirm preferred “vehicles,” they provide hypotheses consistent with known co-selection mechanisms observed across natural and industrial contexts (90–92).

Cladogram–histogram analyses further resolved the genomic relationships of strains TP30 and TR100 within environmental clusters (Fig. 5). Strain TP30 clustered with nine environmental isolates characterized by low ARG abundance and streamlined resistance repertoires (Fig. 5A). Within this cluster, TP30 closely resembled *Pseudomonas* sp. WCS374; both harbored the *mer* operon and displayed higher MRG and phage gene counts than neighboring isolates. Conversely, strain TR100 grouped with *mer-*negative environmental isolates (MS14 and FL1230S1D0), exhibiting low counts of MRGs, ARGs, and MGEs (Fig. 5B).

**Fig 6:**
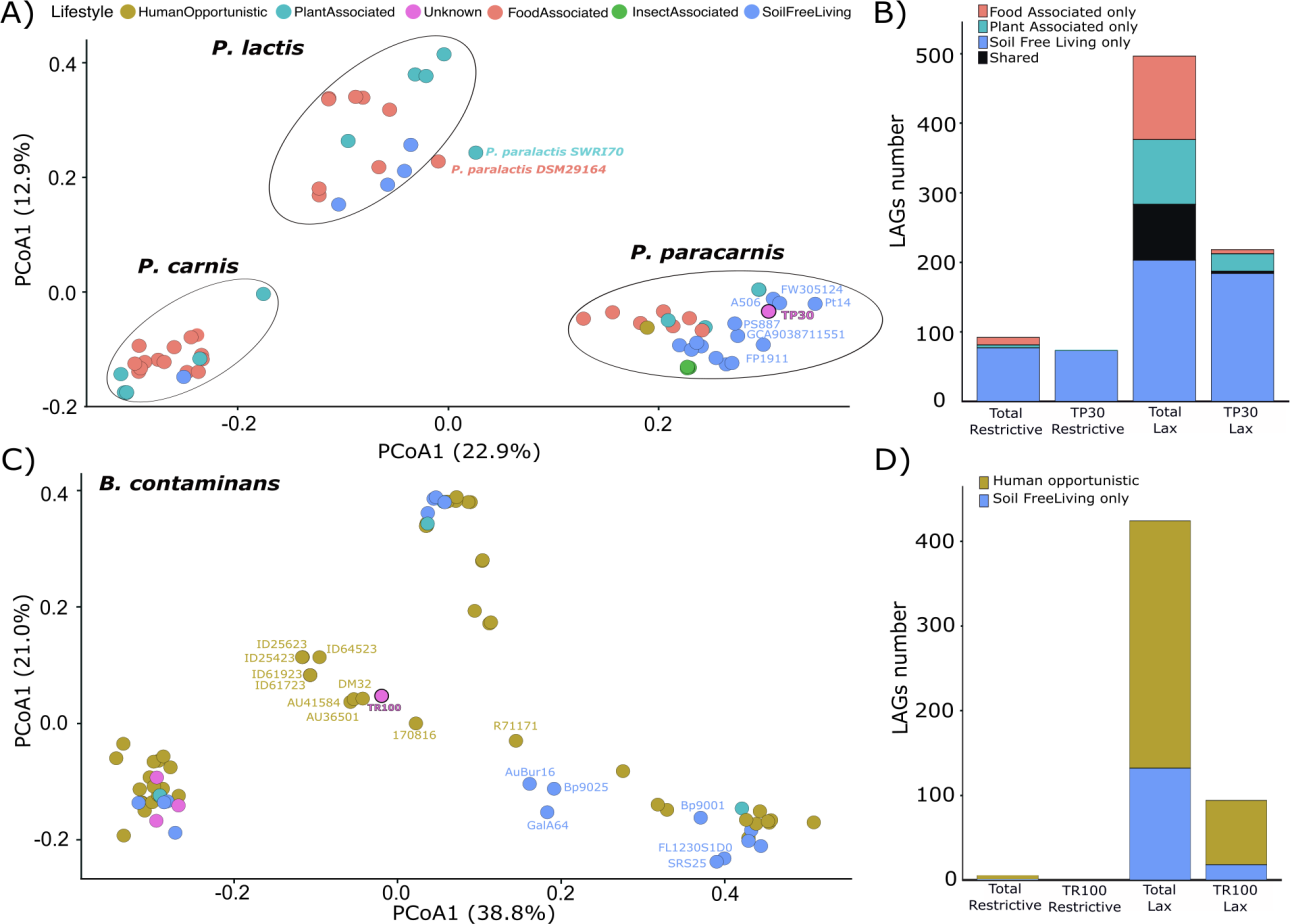
Genome-wide lifestyle patterns and distribution of lifestyle-associated genes (LAGs). (**A**) Principal coordinate analysis (PCoA) of *Pseudomonas* genomes based on the presence–absence matrix that bacLIFE used for lifestyle prediction (Bray–Curtis distances; the first two axes explain 22.9% and 12.9% of total variance). Points are colored by predicted lifestyle (legend, top left). Ellipses highlight the three species groups present in the data set (*P. lactis*, *P. carnis*, and *P. paracarnis*). (**B**) Number of lifestyle-associated genes identified in the *Pseudomonas* data set under two stringency cutoffs: restrictive (genes present in ≥60% of the genomes of a given lifestyle) and lax (genes present in ≥40%). (**C**) PCoA of *Burkholderia contaminans* genomes (axes explain 28.8% and 21.0% of variance). (**D**) Restrictive and lax LAG counts for the *B. contaminans* data set.

To contextualize these co-selection signatures within broader ecological strategies, we next investigated whether the genomic features of TP30 and TR100 aligned with specific ecological lifestyles. Using the bacLIFE framework (21), we inferred lifestyle probabilities and LAGs based on genome-wide orthologous gene-family presence–absence. These predictions were supported by strong out-of-sample discrimination measured with ROC–AUC (AUC = 1: perfect; AUC = 0.5: random). *P. paracarnis* TP30 was predicted primarily as soil free-living (64%, AUC = 0.94 ± 0.05; Fig. S10A), with secondary probabilities for plant-associated (30%, AUC = 0.73 ± 0.15; Fig. S10B) and food-associated (12%, AUC = 0.91 ± 0.05; Fig. S10B) lifestyles, whereas *B. contaminans* TR100 showed its highest probability for a human-opportunistic lifestyle (66%, AUC = 0.88 ± 0.07; Fig. S11B) while retaining a substantial soil free-living signal (32%, AUC = 0.88 ± 0.12; Fig. S11A). Consistent with these predictions, PERMANOVA on Bray–Curtis distances indicated that lifestyle explained a significant fraction of the variance in *Pseudomonas* spp. (*R*^2^ = 0.17, *P* = 0.001; Fig. 6A), reflecting niche-driven diversification and partitioning of lifestyle-associated traits within the genus (86). In contrast, lifestyle did not explain gene-content variation in *B. contaminans* (*R*^2^ = 0.05, *P* = 0.236; Fig. 6C), consistent with the high plasticity and generalist nature described for members of the *B. cepacia* complex (93, 94).

Analysis of lifestyle-associated genes further reinforced these patterns. In *Pseudomonas*, 92 restrictive LAGs were identified, 84% of which were associated with the soil free-living lifestyle, and 95% of these were present in TP30 (Fig. 6B). These genes included siderophore biosynthesis and transport systems, amino acid and organic acid metabolism enzymes, redox homeostasis components, and regulatory elements previously linked to persistence in nutrient-limited or metal-impacted soils (95–102) (Table S9). In contrast, *B. contaminans* displayed fewer restrictive markers predominantly associated with a human-opportunistic lifestyle, none of which were present in TR100 (Fig. 6D). Importantly, TR100 lacked hallmark virulence determinants of pathogenic *B. cepacia* complex strains, supporting its classification as an environmentally adapted, non-pathogenic lineage despite its mixed ecological signal (94), with annotated lax LAGs largely related to genome plasticity, stress persistence, and metal tolerance (Table S10).

Altogether, these analyses show that strong selective pressures in metal-rich sediments shape resistance and mobility gene repertoires toward low ARG abundance and enrichment of MRGs, coupled with distinct mobilization strategies. Within this framework, TP30 emerges as a metabolically versatile, soil-adapted *Pseudomonas* well suited for persistence in contaminated sediments, whereas TR100 represents a genomically plastic yet environmentally adapted *Burkholderia* with limited predicted pathogenic potential. These patterns indicate that co-selection of mercury resistance through mobile genetic elements supports adaptive evolution in hostile environments while remaining decoupled from clinically relevant resistance, reinforcing the suitability of both strains as safe and ecologically coherent candidates for bioremediation applications.

### Conclusion

In conclusion, *Pseudomonas paracarnis* TP30 and *Burkholderia contaminans* TR100 harbor broad-spectrum *mer* operons embedded within mobile genomic contexts that reflect lineage-specific evolutionary trajectories rather than isolation source. Genome-wide comparisons revealed open pangenomes and high plasticity, yet environmental pressures were more clearly reflected in the distribution of specific resistance and mobility determinants than in overall genomic structure. Distinct mobilization strategies emerged, with phage-associated signatures predominating in TP30 and plasmid-embedded modules in TR100, highlighting alternative pathways for the dissemination of mercury resistance and co-selection. Lifestyle predictions further positioned TP30 as a soil-adapted free-living bacterium and TR100 as a genomically plastic but environmentally coherent lineage lacking hallmark virulence determinants. Together, these findings integrate resistance architecture, mobility dynamics, and ecological context, supporting both strains as environmentally adapted and low-risk candidates for further evaluation in mercury bioremediation of mining-impacted Amazonian habitats.

## Data Availability

Genome sequences are available in the NCBI GenBank with the BioProject number PRJNA1445321, and the accession numbers SAMN56786084 and SAMN56786085 for *P. paracarnis* TP30 and *B. contaminans* TR100, respectively.

## SUPPLEMENTAL MATERIAL

Supplemental material spectrum.03127-25-s0001.pdf Fig. S1-S11; Tables S1 to S4, S7 to S10.

Table S5 spectrum.03127-25-s0002.pdf NIb obtained from the comparison of the genome of strain TP30 with type strains (*P. carnis* [T], *P. paracarnis* V5/DAB/2/5 [T], *P. lactis* DSM 29167 [T], and *P. paralactis* DSM 29164 [T]) and 70 genomes of *Pseudomonas* spp.

Table S6 spectrum.03127-25-s0003.xlsx ANIb obtained from the comparison of the genome of strain TR100 with type strain *B. contaminans* LMG 23361 [T] and 79 genomes of *B. contaminans*.

ASM does not own the copyrights to Supplemental Material that may be linked to, or accessed through, an article. The authors have granted ASM a non-exclusive, world-wide license to publish the Supplemental Material files. Please contact the corresponding author directly for reuse.

## References

[1] Moreno-Brush M, McLagan DS, Biester H. 2020. Fate of mercury from artisanal and small-scale gold mining in tropical rivers: hydrological and biogeochemical controls. A critical review. Crit Rev Environ Sci Technol 50:437–475. doi:10.1080/10643389.2019.1629793

[2] UNEP. 2020. Artisanal and Small-Scale Gold Mining (ASGM). Available from: https://www.unep.org/globalmercurypartnership/what-we-do/artisanal-and-small-scale-gold-mining-asgm

[3] Yu R-Q, Barkay T. 2022. Microbial mercury transformations: molecules, functions and organisms. Adv Appl Microbiol 118:31–90. doi:10.1016/bs.aambs.2022.03.001 35461663

[4] Egbo TE, Dickson JO, Miller C, Johs A, Sanders CA, Robertson BK. 2017. Characterization, identification and seasonal evaluation of microbes in mercury contaminated soils. Front Sci Technol Eng Math 1:15–26.

[5] Kumar V, Umesh M, Shanmugam MK, Chakraborty P, Duhan L, Gummadi SN, Pasrija R, Jayaraj I, Dasarahally Huligowda LK. 2023. A retrospection on mercury contamination, bioaccumulation, and toxicity in diverse environments: current insights and future prospects. Sustainability 15:13292. doi:10.3390/su151813292

[6] Canham R, González-Prieto AM, Elliott JE. 2021. Mercury exposure and toxicological consequences in fish and fish-eating wildlife from anthropogenic activity in Latin America. Integr Environ Assess Manag 17:13–26. doi:10.1002/ieam.4313 32662936 PMC7821190

[7] Boyd ES, Barkay T. 2012. The mercury resistance operon: from an origin in a geothermal environment to an efficient detoxification machine. Front Microbio 3:349. doi:10.3389/fmicb.2012.00349 PMC3466566 23087676

[8] Matsui K, Endo G. 2018. Mercury bioremediation by mercury resistance transposon-mediated in situ molecular breeding. Appl Microbiol Biotechnol 102:3037–3048. doi:10.1007/s00253-018-8847-2 29479648

[9] Kumari S, Jamwal R, Mishra N, Singh DK, Amit JR. 2020. Recent developments in environmental mercury bioremediation and its toxicity: a review. Environ Nanotechnol Monit Manag 13:100283. doi:10.1016/j.enmm.2020.100283

[10] Gillieatt BF, Coleman NV. 2024. Unravelling the mechanisms of antibiotic and heavy metal resistance co-selection in environmental bacteria. FEMS Microbiol Rev 48:fuae017. doi:10.1093/femsre/fuae017 38897736 PMC11253441

[11] Murray LM, Hayes A, Snape J, Kasprzyk-Hordern B, Gaze WH, Murray AK. 2024. Co-selection for antibiotic resistance by environmental contaminants. NPJ Antimicrob Resist 2:9. doi:10.1038/s44259-024-00026-7 39843965 PMC11721650

[12] Cunningham CJ, Kuyukina MS, Ivshina IB, Konev AI, Peshkur TA, Knapp CW. 2020. Potential risks of antibiotic resistant bacteria and genes in bioremediation of petroleum hydrocarbon contaminated soils. Environ Sci Process Impacts 22:1110–1124. doi:10.1039/c9em00606k 32236187

[13] Li X, Yang Z, Zhang G, Si S, Wu X, Cai L. 2022. Plasmid genomes reveal the distribution, abundance, and organization of mercury-related genes and their co-distribution with antibiotic resistant genes in Gammaproteobacteria. Genes (Basel) 13:2149. doi:10.3390/genes13112149 36421823 PMC9690531

[14] Kothari A, Soneja D, Tang A, Carlson HK, Deutschbauer AM, Mukhopadhyay A. 2019. Native plasmid-encoded mercury resistance genes are functional and demonstrate natural transformation in environmental bacterial isolates. mSystems 4:10.1128/msystems.00588-19. doi:10.1128/mSystems.00588-19 PMC6918032 31848306

[15] Pu Q, Zhang K, Poulain AJ, Liu J, Zhang R, Abdelhafiz MA, Meng B, Feng X. 2022. Mercury drives microbial community assembly and ecosystem multifunctionality across a Hg contamination gradient in rice paddies. J Hazard Mater 435:129055. doi:10.1016/j.jhazmat.2022.129055 35650726

[16] Cardona GI, Escobar MC, Acosta-González A, Díaz-Ruíz N, Niño-García JP, Vasquez Y, Marrugo-Negrete J, Marqués S. 2024. Microbial diversity and abundance of Hg related genes from water, sediment and soil the Colombian amazon ecosystems impacted by artisanal and small-scale gold mining. Chemosphere 352:141348. doi:10.1016/j.chemosphere.2024.141348 38340998

[17] Lemos LN, Pedrinho A, Vasconcelos ATR de, Tsai SM, Mendes LW. 2021. Amazon deforestation enriches antibiotic resistance genes. Soil Biol Biochem 153:108110. doi:10.1016/j.soilbio.2020.108110

[18] Thompson CC, Tschoeke D, Coutinho FH, Leomil L, Garcia GD, Otsuki K, Turcq BJ, Moreira LS, Turcq PFM, Cordeiro RC, Asp NE, Thompson FL. 2023. Diversity of microbiomes across a 13,000-year-old Amazon sediment. Microb Ecol 86:2202–2209. doi:10.1007/s00248-023-02202-0 37017718

[19] Christakis CA, Barkay T, Boyd ES. 2021. Expanded diversity and phylogeny of mer genes broadens mercury resistance paradigms and reveals an origin for MerA among thermophilic archaea. Front Microbiol 12:682605. doi:10.3389/fmicb.2021.682605 34248899 PMC8261052

[20] Singh BK, Jiang G, Wei Z, Sáez-Sandino T, Gao M, Liu H, Xiong C. 2025. Plant pathogens, microbiomes, and soil health. Trends Microbiol 33:887–902. doi:10.1016/j.tim.2025.03.013 40274492

[21] Guerrero-Egido G, Pintado A, Bretscher KM, Arias-Giraldo LM, Paulson JN, Spaink HP, Claessen D, Ramos C, Cazorla FM, Medema MH, Raaijmakers JM, Carrión VJ. 2024. bacLIFE: a user-friendly computational workflow for genome analysis and prediction of lifestyle-associated genes in bacteria. Nat Commun 15:2072. doi:10.1038/s41467-024-46302-y 38453959 PMC10920822

[22] Mahenthiralingam E, Baldwin A, Dowson CG. 2008. Burkholderia cepacia complex bacteria: opportunistic pathogens with important natural biology. J Appl Microbiol 104:1539–1551. doi:10.1111/j.1365-2672.2007.03706.x 18217926

[23] Compant S, Nowak J, Coenye T, Clément C, Ait Barka E. 2008. Diversity and occurrence of Burkholderia spp. in the natural environment. FEMS Microbiol Rev 32:607–626. doi:10.1111/j.1574-6976.2008.00113.x 18422616

[24] Sessitsch A, Coenye T, Sturz AV, Vandamme P, Barka EA, Salles JF, Van Elsas JD, Faure D, Reiter B, Glick BR, Wang-Pruski G, Nowak J. 2005. Burkholderia phytofirmans sp. nov., a novel plant-associated bacterium with plant-beneficial properties. Int J Syst Evol Microbiol 55:1187–1192. doi:10.1099/ijs.0.63149-0 15879253

[25] Wang D, Lin H, Shan Y, Song J, Zhang DD, Dai XF, Han D, Chen JY. 2024. The potential of Burkholderia gladioli KRS027 in plant growth promotion and biocontrol against Verticillium dahliae revealed by dual transcriptome of pathogen and host. Microbiol Res 287:127836. doi:10.1016/j.micres.2024.127836 39018831

[26] Li X, Gu N, Huang TY, Zhong F, Peng G. 2023. Pseudomonas aeruginosa: a typical biofilm forming pathogen and an emerging but underestimated pathogen in food processing. Front Microbiol 13:1114199. doi:10.3389/fmicb.2022.1114199 36762094 PMC9905436

[27] Abdullahi IN, Mejri S, Okwume CC, Lawal NA, Olusegun OA, Sallem RB, Slama KB. 2025. Global epidemiology of high priority and pandemic Pseudomonas aeruginosa in pets, livestock, wild, and aquatic animals: a systematic review and meta-analysis. Lett Appl Microbiol 78:vaf028. doi:10.1093/lambio/ovaf028 39999856

[28] Yang P, Zhao L, Gao YG, Xia Y. 2023. Detection, diagnosis, and preventive management of the bacterial plant pathogen Pseudomonas syringae. Plants (Basel) 12:1765. doi:10.3390/plants12091765 37176823 PMC10181079

[29] Janaki M, Kirupanantha-Rajan P, Senthil-Nathan S, Stanley-Raja V, Al Farraj DA, Aljeidi RA, Arokiyaraj S. 2024. Beneficial role of Burkholderia cepacia in heavy metal bioremediation in metal-polluted soils and enhance the tomato plant growth. Biocatal Agric Biotechnol 57:103032. doi:10.1016/j.bcab.2024.103032

[30] Chellaiah ER. 2018. Cadmium (heavy metals) bioremediation by Pseudomonas aeruginosa: a minireview. Appl Water Sci 8:154. doi:10.1007/s13201-018-0796-5

[31] Cardona GI, Escobar MC, Acosta-González A, Marín P, Marqués S. 2022. Highly mercury-resistant strains from different Colombian Amazon ecosystems affected by artisanal gold mining activities. Appl Microbiol Biotechnol 106:2775–2793. doi:10.1007/s00253-022-11860-y 35344092 PMC8990959

[32] Zhang W, Chen L, Liu D. 2012. Characterization of a marine-isolated mercury-resistant Pseudomonas putida strain SP1 and its potential application in marine mercury reduction. Appl Microbiol Biotechnol 93:1305–1314. doi:10.1007/s00253-011-3454-5 21751007

[33] François F, Lombard C, Guigner JM, Soreau P, Brian-Jaisson F, Martino G, Vandervennet M, Garcia D, Molinier AL, Pignol D, Peduzzi J, Zirah S, Rebuffat S. 2012. Isolation and characterization of environmental bacteria capable of extracellular biosorption of mercury. Appl Environ Microbiol 78:1097–1106. doi:10.1128/AEM.06522-11 22156431 PMC3273009

[34] Mehdi Kargar. 2012. Identification and molecular analysis of mercury resistant bacteria in Kor River, Iran. Afr J Biotechnol 11:6710–6717. doi:10.5897/AJB11.3394

[35] Santos-Gandelman JF, Giambiagi-deMarval M, Muricy G, Barkay T, Laport MS. 2014. Mercury and methylmercury detoxification potential by sponge-associated bacteria. Antonie Van Leeuwenhoek 106:585–590. doi:10.1007/s10482-014-0224-2 24996548

[36] Chang J, Yan Z, Dong J, Wu X, Meng Z, Shi Y, Chen J. 2022. Mechanisms controlling the transformation of and resistance to mercury(II) for a plant-associated Pseudomonas sp. strain, AN-B15. J Hazard Mater 425:127948. doi:10.1016/j.jhazmat.2021.127948 34915295

[37] Leger A, Leonardi T. 2019. pycoQC, interactive quality control for Oxford Nanopore Sequencing. J Open Source Softw 4:1236. doi:10.21105/joss.01236

[38] Chen Y, Nie F, Xie S-Q, Zheng Y-F, Dai Q, Bray T, Wang Y-X, Xing J-F, Huang Z-J, Wang D-P, He L-J, Luo F, Wang J-X, Liu Y-Z, Xiao C-L. 2021. Efficient assembly of nanopore reads via highly accurate and intact error correction. Nat Commun 12:60. doi:10.1038/s41467-020-20236-7 33397900 PMC7782737

[39] Kolmogorov M, Yuan J, Lin Y, Pevzner PA. 2019. Assembly of long, error-prone reads using repeat graphs. Nat Biotechnol 37:540–546. doi:10.1038/s41587-019-0072-8 30936562

[40] Koren S, Walenz BP, Berlin K, Miller JR, Bergman NH, Phillippy AM. 2017. Canu: scalable and accurate long-read assembly via adaptive k-mer weighting and repeat separation. Genome Res 27:722–736. doi:10.1101/gr.215087.116 28298431 PMC5411767

[41] Gonzalez-Garcia L, Guevara-Barrientos D, Lozano-Arce D, Gil J, Díaz-Riaño J, Duarte E, Andrade G, Bojacá JC, Hoyos-Sanchez MC, Chavarro C, Guayazan N, Chica LA, Buitrago Acosta MC, Bautista E, Trujillo M, Duitama J. 2023. New algorithms for accurate and efficient de novo genome assembly from long DNA sequencing reads. Life Sci Alliance 6:e202201719. doi:10.26508/lsa.202201719 36813568 PMC9946810

[42] Gurevich A, Saveliev V, Vyahhi N, Tesler G. 2013. QUAST: quality assessment tool for genome assemblies. Bioinformatics 29:1072–1075. doi:10.1093/bioinformatics/btt086 23422339 PMC3624806

[43] Seppey M, Manni M, Zdobnov EM. 2019. BUSCO: assessing genome assembly and annotation completeness, p 227–245. *In* Kollmar M (ed), Gene prediction: methods and protocols. Springer, New York, New York, NY. 10.1007/978-1-4939-9173-0_14 31020564

[44] Aziz RK, Bartels D, Best AA, DeJongh M, Disz T, Edwards RA, Formsma K, Gerdes S, Glass EM, Kubal M, et al.. 2008. The RAST Server: rapid annotations using subsystems technology. BMC Genomics 9:75. doi:10.1186/1471-2164-9-75 18261238 PMC2265698

[45] Seemann T. 2014. Prokka: rapid prokaryotic genome annotation. Bioinformatics 30:2068–2069. doi:10.1093/bioinformatics/btu153 24642063

[46] Alcock BP, Huynh W, Chalil R, Smith KW, Raphenya AR, Wlodarski MA, Edalatmand A, Petkau A, Syed SA, Tsang KK, et al.. 2023. CARD 2023: expanded curation, support for machine learning, and resistome prediction at the Comprehensive Antibiotic Resistance Database. Nucleic Acids Res 51:D690–D699. doi:10.1093/nar/gkac920 36263822 PMC9825576

[47] Arango-Argoty G, Garner E, Pruden A, Heath LS, Vikesland P, Zhang L. 2018. DeepARG: a deep learning approach for predicting antibiotic resistance genes from metagenomic data. Microbiome 6:23. doi:10.1186/s40168-018-0401-z 29391044 PMC5796597

[48] Cumsille A, Durán RE, Rodríguez-Delherbe A, Saona-Urmeneta V, Cámara B, Seeger M, Araya M, Jara N, Buil-Aranda C. 2023 GenoVi, an open-source automated circular genome visualizer for bacteria and archaea. PLoS Comput Biol 19:e1010998. doi:10.1371/journal.pcbi.1010998 37014908 PMC10104344

[49] Grant JR, Enns E, Marinier E, Mandal A, Herman EK, Chen C, Graham M, Van Domselaar G, Stothard P. 2023. Proksee: in-depth characterization and visualization of bacterial genomes. Nucleic Acids Res 51:W484–W492. doi:10.1093/nar/gkad326 37140037 PMC10320063

[50] Glaeser SP, Kämpfer P. 2015. Multilocus sequence analysis (MLSA) in prokaryotic taxonomy. Syst Appl Microbiol 38:237–245. doi:10.1016/j.syapm.2015.03.007 25959541

[51] Richter M, Rosselló-Móra R, Oliver Glöckner F, Peplies J. 2016. JSpeciesWS: a web server for prokaryotic species circumscription based on pairwise genome comparison. Bioinformatics 32:929–931. doi:10.1093/bioinformatics/btv681 26576653 PMC5939971

[52] Löytynoja A, Goldman N. 2010. webPRANK: a phylogeny-aware multiple sequence aligner with interactive alignment browser. BMC Bioinformatics 11:579. doi:10.1186/1471-2105-11-579 21110866 PMC3009689

[53] Vaughan TG. 2017. IcyTree: rapid browser-based visualization for phylogenetic trees and networks. Bioinformatics 33:2392–2394. doi:10.1093/bioinformatics/btx155 28407035 PMC5860111

[54] Gautreau G, Bazin A, Gachet M, Planel R, Burlot L, Dubois M, Perrin A, Médigue C, Calteau A, Cruveiller S, Matias C, Ambroise C, Rocha EPC, Vallenet D. 2020. PPanGGOLiN: depicting microbial diversity via a partitioned pangenome graph. PLoS Comput Biol 16:e1007732. doi:10.1371/journal.pcbi.1007732 32191703 PMC7108747

[55] R Core Team. 2013. R: a language and environment for statistical computing. Foundations of Statistical Computing, Vienna, Austria.

[56] Allaire J. 2012. RStudio: integrated development environment for R. RStudio, Boston, MA.

[57] Gu Z, Hübschmann D. 2022. Make interactive complex heatmaps in R. Bioinformatics 38:1460–1462. doi:10.1093/bioinformatics/btab806 34864868 PMC8826183

[58] Harrell Jr FE. 2019. Package ‘hmisc.’ CRAN2018

[59] Oksanen J, Blanchet FG, Kindt R, Legendre P, Minchin PR, O’hara RB, Simpson GL, Solymos P, Stevens MHH, Wagner H. 2013. Package ‘vegan’. Community ecology package

[60] Wilkins D, Kurtz Z. 2019. gggenes: draw gene arrow maps in ‘ggplot2’R package version 0.4. 0

[61] Bochkareva OO, Moroz EV, Davydov II, Gelfand MS. 2018. Genome rearrangements and selection in multi-chromosome bacteria Burkholderia spp. BMC Genomics 19:965. doi:10.1186/s12864-018-5245-1 30587126 PMC6307245

[62] Engin AB, Engin ED, Engin A. 2023. Effects of co-selection of antibiotic-resistance and metal-resistance genes on antibiotic-resistance potency of environmental bacteria and related ecological risk factors. Environ Toxicol Pharmacol 98:104081. doi:10.1016/j.etap.2023.104081 36805463

[63] You LX, Zhang RR, Dai JX, Lin ZT, Li YP, Herzberg M, Zhang JL, Al-Wathnani H, Zhang CK, Feng RW, Liu H, Rensing C. 2021. Potential of cadmium resistant Burkholderia contaminans strain ZCC in promoting growth of soy beans in the presence of cadmium. Ecotoxicol Environ Saf 211:111914. doi:10.1016/j.ecoenv.2021.111914 33454593

[64] Lan Y, Liu M, Song Y, Cao Y, Li F, Luo D, Qiao D. 2023. Distribution, characterization, and evolution of heavy metal resistance genes and Tn7-like associated heavy metal resistance Gene Island of Burkholderia. Front Microbiol 14:1252127. doi:10.3389/fmicb.2023.1252127 38075907 PMC10702557

[65] diCenzo GC, Finan TM. 2017. The divided bacterial genome: structure, function, and evolution. Microbiol Mol Biol Rev 81:10–1128. doi:10.1128/MMBR.00019-17 PMC5584315 28794225

[66] Harrison PW, Lower RPJ, Kim NKD, Young JPW. 2010. Introducing the bacterial ‘chromid’: not a chromosome, not a plasmid. Trends Microbiol 18:141–148. doi:10.1016/j.tim.2009.12.010 20080407

[67] Trahan C, Pandey RS, Singh U, Choudhary A, Cho H, Azad RK, Choudhary M. 2019. Multiple chromosomes in bacteria: low level of evolutionary constraint drives the rapid genetic divergence of chromosome II. Adv Microbiol 09:656–677. doi:10.4236/aim.2019.97041

[68] Carroll AC, Wong A. 2018. Plasmid persistence: costs, benefits, and the plasmid paradox. Can J Microbiol 64:293–304. doi:10.1139/cjm-2017-0609 29562144

[69] Gullberg E, Albrecht LM, Karlsson C, Sandegren L, Andersson DI. 2014. Selection of a multidrug resistance plasmid by sublethal levels of antibiotics and heavy metals. mBio 5:10.1128/mbio.01918-14. doi:10.1128/mBio.01918-14 PMC4196238 25293762

[70] Rodríguez-Beltrán J, DelaFuente J, León-Sampedro R, MacLean RC, San Millán Á. 2021. Beyond horizontal gene transfer: the role of plasmids in bacterial evolution. Nat Rev Microbiol 19:347–359. doi:10.1038/s41579-020-00497-1 33469168

[71] Naguib MM, El-Gendy AO, Khairalla AS. 2018. Microbial diversity of mer operon genes and their potential rules in mercury bioremediation and resistance. Open Biotechnol J 12:56–77. doi:10.2174/1874070701812010056

[72] Mergeay M, Houdt R. 2015. Metal response in Cupriavidus metallidurans: volume I: from habitats to genes and proteins. *In* Volume I: From Habitats to Genes and Proteins. Springer.

[73] Perron K, Caille O, Rossier C, Van Delden C, Dumas J-L, Köhler T. 2004. CzcR-CzcS, a two-component system involved in heavy metal and carbapenem resistance in Pseudomonas aeruginosa. J Biol Chem 279:8761–8768. doi:10.1074/jbc.M312080200 14679195

[74] Agarwal M, Rathore RS, Jagoe C, Chauhan A. 2019. Multiple lines of evidences reveal mechanisms underpinning mercury resistance and volatilization by Stenotrophomonas sp. MA5 isolated from the savannah river site (SRS), USA. Cells 8:309. doi:10.3390/cells8040309 30987227 PMC6523443

[75] Christensen GA, Gionfriddo CM, King AJ, Moberly JG, Miller CL, Somenahally AC, Callister SJ, Brewer H, Podar M, Brown SD, Palumbo AV, Brandt CC, Wymore AM, Brooks SC, Hwang C, Fields MW, Wall JD, Gilmour CC, Elias DA. 2019. Determining the reliability of measuring mercury cycling gene abundance with correlations with mercury and methylmercury concentrations. Environ Sci Technol 53:8649–8663. doi:10.1021/acs.est.8b06389 31260289

[76] Parks JM, Johs A, Podar M, Bridou R, Hurt RA Jr, Smith SD, Tomanicek SJ, Qian Y, Brown SD, Brandt CC, Palumbo AV, Smith JC, Wall JD, Elias DA, Liang L. 2013. The genetic basis for bacterial mercury methylation. Science 339:1332–1335. doi:10.1126/science.1230667 23393089

[77] Schaefer JK, Kronberg RM, Morel FMM, Skyllberg U. 2014. Detection of a key Hg methylation gene, hgcA, in wetland soils. Environ Microbiol Rep 6:441–447. doi:10.1111/1758-2229.12136 25646534

[78] Pal C, Asiani K, Arya S, Rensing C, Stekel DJ, Larsson DJ, Hobman JL. 2017. Chapter seven - Metal resistance and its association with antibiotic resistance, p 261–313. *In* Poole RK (ed), Microbiology of Metal Ions. Academic Press. 10.1016/bs.ampbs.2017.02.001 28528649

[79] Costa SS, Guimarães LC, Silva A, Soares SC, Baraúna RA. 2020. First steps in the analysis of prokaryotic pan-genomes. Bioinform Biol Insights 14:1177932220938064. doi:10.1177/1177932220938064 32843837 PMC7418249

[80] Spring-Pearson SM, Stone JK, Doyle A, Allender CJ, Okinaka RT, Mayo M, Broomall SM, Hill JM, Karavis MA, Hubbard KS, Insalaco JM, McNew LA, Rosenzweig CN, Gibbons HS, Currie BJ, Wagner DM, Keim P, Tuanyok A. 2015. Pangenome analysis of Burkholderia pseudomallei: genome evolution preserves gene order despite high recombination rates. PLoS One 10:e0140274. doi:10.1371/journal.pone.0140274 26484663 PMC4613141

[81] Hall JPJ, Harrison E, Pärnänen K, Virta M, Brockhurst MA. 2020. The impact of mercury selection and conjugative genetic elements on community structure and resistance gene transfer. Front Microbiol 11:1846. doi:10.3389/fmicb.2020.01846 32849443 PMC7419628

[82] Ready D, Pratten J, Mordan N, Watts E, Wilson M. 2007. The effect of amalgam exposure on mercury- and antibiotic-resistant bacteria. Int J Antimicrob Agents 30:34–39. doi:10.1016/j.ijantimicag.2007.02.009 17459664

[83] Saha DK, Ghosh S, Chaudhuri J, Mandal A. 2006. Mercury resistance in bacterial strains isolated from hospitals and clinics. Bull Environ Contam Toxicol 77:88–95. doi:10.1007/s00128-006-1036-5 16832760

[84] Wang H, Xia F, Xia Y, Li J, Hu Y, Deng Y, Zou M. 2024. Pangenome analysis of Shewanella xiamenensis revealed important genetic traits concerning genetic diversity, pathogenicity and antibiotic resistance. BMC Genomics 25:216. doi:10.1186/s12864-024-10146-z 38413855 PMC10898099

[85] Yu Z, Gunn L, Wall P, Fanning S. 2017. Antimicrobial resistance and its association with tolerance to heavy metals in agriculture production. Food Microbiol 64:23–32. doi:10.1016/j.fm.2016.12.009 28213031

[86] Sharma A, Sangwan N, Negi V, Kohli P, Khurana JP, Rao DLN, Lal R. 2015. Pan-genome dynamics of Pseudomonas gene complements enriched across hexachlorocyclohexane dumpsite. BMC Genomics 16:313. doi:10.1186/s12864-015-1488-2 25898829 PMC4405911

[87] Middelboe M, Traving SJ, Castillo D, Kalatzis PG, Glud RN. 2025. Prophage-encoded chitinase gene supports growth of its bacterial host isolated from deep-sea sediments. ISME J 19:wraf004. doi:10.1093/ismejo/wraf004 39832281 PMC11788074

[88] Vandecraen J, Chandler M, Aertsen A, Van Houdt R. 2017. The impact of insertion sequences on bacterial genome plasticity and adaptability. Crit Rev Microbiol 43:709–730. doi:10.1080/1040841X.2017.1303661 28407717

[89] Yao Y, Maddamsetti R, Weiss A, Ha Y, Wang T, Wang S, You L. 2022. Intra- and interpopulation transposition of mobile genetic elements driven by antibiotic selection. Nat Ecol Evol 6:555–564. doi:10.1038/s41559-022-01705-2 35347261 PMC12520065

[90] Song J, Rensing C, Holm PE, Virta M, Brandt KK. 2017. Comparison of metals and tetracycline as selective agents for development of tetracycline resistant bacterial communities in agricultural soil. Environ Sci Technol 51:3040–3047. doi:10.1021/acs.est.6b05342 28198616

[91] Icgen B, Yilmaz F. 2014. Co-occurrence of antibiotic and heavy metal resistance in Kızılırmak River isolates. Bull Environ Contam Toxicol 93:735–743. doi:10.1007/s00128-014-1383-6 25257221

[92] Zhao R, Yu K, Zhang J, Zhang G, Huang J, Ma L, Deng C, Li X, Li B. 2020. Deciphering the mobility and bacterial hosts of antibiotic resistance genes under antibiotic selection pressure by metagenomic assembly and binning approaches. Water Res 186:116318. doi:10.1016/j.watres.2020.116318 32871290

[93] Eberl L, Vandamme P. 2016. Members of the genus Burkholderia: good and bad guys. F1000Res 5:1007. doi:10.12688/f1000research.8221.1 PMC4882756 27303639

[94] Mahenthiralingam E, Urban TA, Goldberg JB. 2005. The multifarious, multireplicon Burkholderia cepacia complex. Nat Rev Microbiol 3:144–156. doi:10.1038/nrmicro1085 15643431

[95] Ahmed E, Holmström SJM. 2014. Siderophores in environmental research: roles and applications. Microb Biotechnol 7:196–208. doi:10.1111/1751-7915.12117 24576157 PMC3992016

[96] Nascimento FX, Urón P, Glick BR, Giachini A, Rossi MJ. 2021. Genomic analysis of the 1-aminocyclopropane-1-carboxylate deaminase-producing Pseudomonas thivervalensis SC5 reveals its multifaceted roles in soil and in beneficial interactions with plants. Front Microbiol 12:752288. doi:10.3389/fmicb.2021.752288 34659189 PMC8515041

[97] Deguchi Y, Banba M, Shimoda Y, Chechetka SA, Suzuri R, Okusako Y, Ooki Y, Toyokura K, Suzuki A, Uchiumi T, Higashi S, Abe M, Kouchi H, Izui K, Hata S. 2007. Transcriptome profiling of Lotus japonicus roots during arbuscular mycorrhiza development and comparison with that of nodulation. DNA Res 14:117–133. doi:10.1093/dnares/dsm014 17634281 PMC2779901

[98] Dunn MF. 2015. Key roles of microsymbiont amino acid metabolism in rhizobia-legume interactions. Crit Rev Microbiol 41:411–451. doi:10.3109/1040841X.2013.856854 24601835

[99] Coba de la Peña T, Redondo FJ, Fillat MF, Lucas MM, Pueyo JJ. 2013. Flavodoxin overexpression confers tolerance to oxidative stress in beneficial soil bacteria and improves survival in the presence of the herbicides paraquat and atrazine. J Appl Microbiol 115:236–246. doi:10.1111/jam.12224 23594228

[100] Joshi H, Khan A. 2024. Competition-driven phenotypic plasticity in Iron acquisition and aromatic utilization confers a fitness advantage to Pseudomonas putida in an Iron-limited rhizospheric environment. World J Microbiol Biotechnol 40:386. doi:10.1007/s11274-024-04192-8 39565458 PMC11579168

[101] Venturi V, Keel C. 2016. Signaling in the rhizosphere. Trends Plant Sci 21:187–198. doi:10.1016/j.tplants.2016.01.005 26832945

[102] Gagarinova A, Hosseinnia A, Rahmatbakhsh M, Istace Z, Phanse S, Moutaoufik MT, Zilocchi M, Zhang Q, Aoki H, Jessulat M, Kim S, Aly KA, Babu M. 2022. Auxotrophic and prototrophic conditional genetic networks reveal the rewiring of transcription factors in Escherichia coli. Nat Commun 13:4085. doi:10.1038/s41467-022-31819-x 35835781 PMC9283627

