# Supplementary material for: Genome-based insights into metal co-resistance in Amazonian mercury-resistant bacteria: ecological lifestyle and biosafety implications for bioremediation
Source: Microbiol Spectr. 2026 Apr 30;14(6):e03127-25. doi: 10.1128/spectrum.03127-25 (PMC13228018; doi:10.1128/spectrum.03127-25)
Supplement: Supplemental material — Fig. S1-S11; Tables S1 to S4, S7 to S10. [file spectrum.03127-25-s0001.pdf]

SUPPLEMENTARY MATERIAL OF

Genome-based insights into metal co-resistance in Amazonian mercury-resistant bacteria: ecological lifestyle and biosafety implications for bioremediation

Bacterial morphology

We examined *Pseudomonas* sp. TP30 and *Burkholderia* sp.TR100 strains by scanning electron microscopy (SEM) after growth on LB medium at 30ºC for 48 hours. The TP30 strain grew as straight or slightly curved rods (0.45–0.7 × 3.0–1.2-3.0 µm) with rounded edges (Supplementary Figure S1A, SEM), exhibiting a typical gram-negative cell envelope ultrastructure (Supplementary Figure S1C, TEM). Some cells appeared to be in the process of cell division, with symmetric placement of the division septum, with the periplasmic space (P) situated between the outer membrane (O) and cytoplasmic membrane (C). In contrast, the TR100 strain displayed a plump and rod-shaped surface morphology with round edges (Supplementary Figure S1B, SEM) and the typical Gram-negative cell wall ultrastructure, with numerous cytoplasmic inclusion bodies (IB) (Supplementary Figure S1E, TEM), with a smaller size than TP30 (0.7–0.8×1.4 – 1.5 µm) (Supplementary Figure S1B, SEM). These inclusion bodies likely correspond to polyhydroxyalkanoates (PHA), which is a common feature within *Burkholderia* species. (1–3). In addition, there was a slight reduction in inclusion body size when cells were grown in Hg-supplemented cultures. (Supplementary Figure 1G, SEM). In both cases, the cell morphology remained unaltered when cultures were supplemented with mercury. Although some strains have shown evidence of intracellular Hg accumulation (4), we found no evidence of Hg deposits in the intracellular space of TP30 and TR100 (Supplementary Figure S1E and H), suggesting that the mechanism of resistance in both strains does not involve the accumulation and/or precipitation of mercury in the cytoplasm. As shown previously, the mercury reductase activity for Hg volatilization may be the primary mechanism of mercury resistance in these two strains(5).

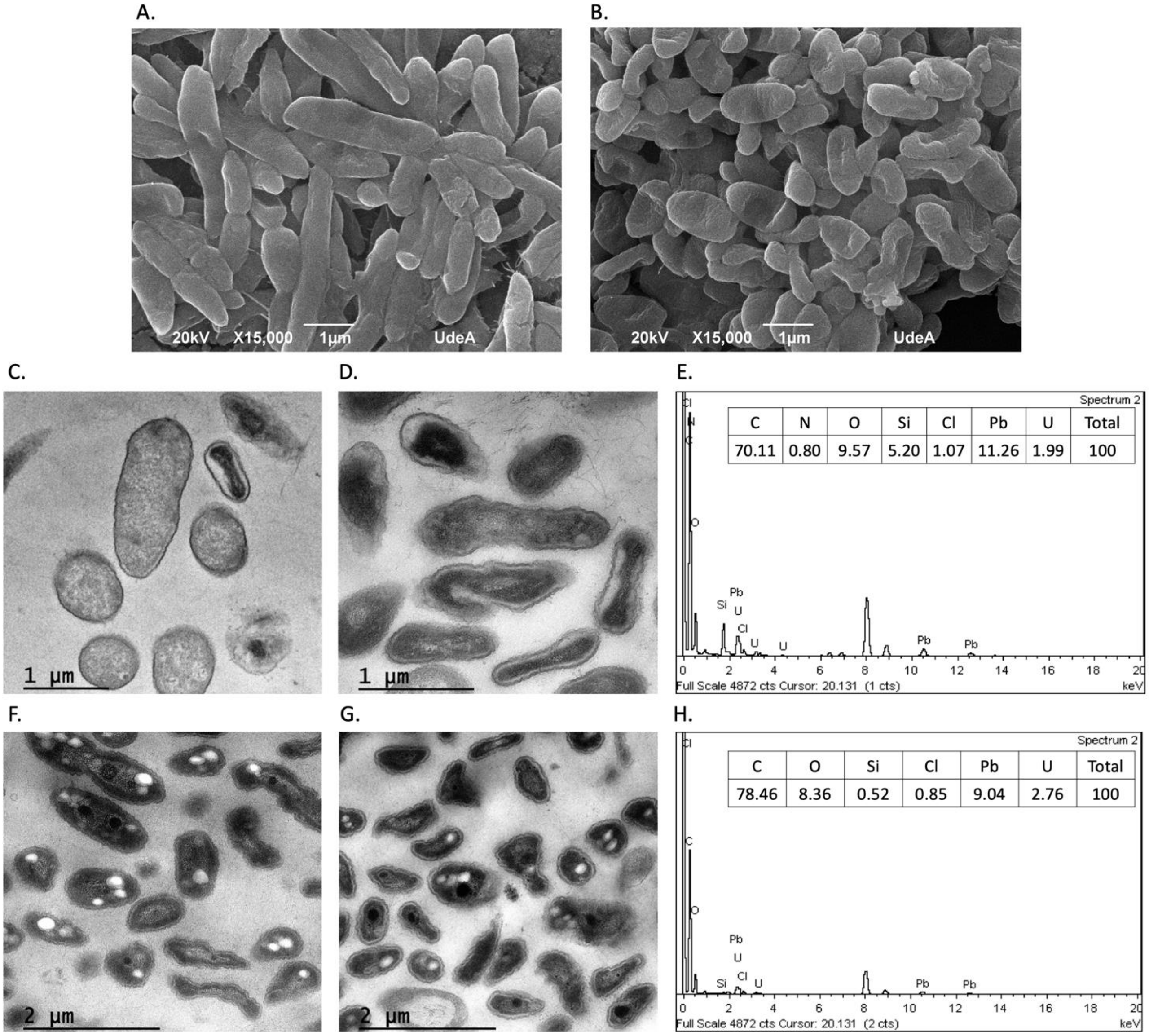

Supplementary Figure S1. Scanning electron microscopy (SEM) and transmission electron microscopy (TEM) images of *Pseudomonas* sp. TP30 grown on Luria-Bertani (LB) in the absence of Hg (SEM at 15,000X, **A**; TEM, **C**) and the presence of 40 mg L^{-1} $HgCl_2$ (TEM, **D**), and of *Burkholderia sp*. TR100 grown on LB in the absence of Hg (SEM at 15,000X, **B**; TEM, **F**) and in the presence of 40 mg L^{-1} $HgCl_2$ (TEM, **G**). The EDS spectrum of TP30 strain **(E)** and TR100 strain **(H)** grown in the presence of 40 mg L^{-1} $HgCl_2$ is presented below. Arrows are used to indicate the presence of different structures in the cells: O = outer membrane; P = periplasmic space; I = inner membrane; IB = inclusion bodies (probably PHB granules).

Sequence quality, genome assembly, and phylogenetic characterization

Illumina sequencing generated 22,408,438 and 13,436,130 raw reads for TP30 and TR100, respectively. Nanopore sequencing produced 26,628 and 39,602 reads for TP30 and TR100, respectively (Supplementary Table S1). Between 84% and 99% of the reads passed the quality control filters (Supplementary Figure S2, see methods for details).

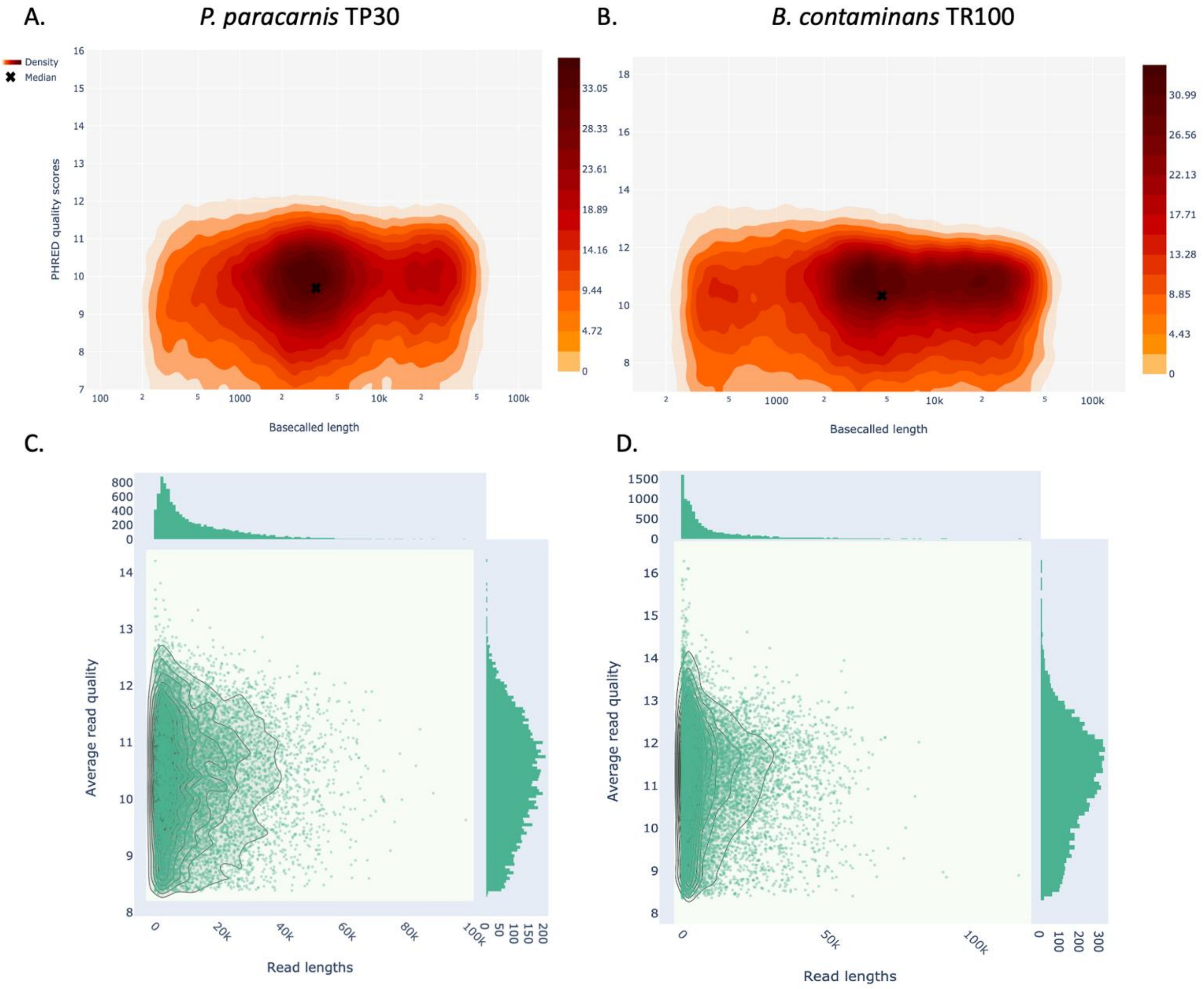

Supplementary Figure S2. PHRED quality scores of **A)** TP30 and **B)** TR100 Nanopore reads. Average read quality vs. read lengths of **C)** TP30 and **D)** TR100 Nanopore reads.

The bacterial genomes were initially assembled based on the Nanopore sequences, followed by a polishing process using Illumina sequencing reads (see methods for details). For the *P. paracarnis* TP30, a single contig was consistently obtained with all the assemblers tested (Flye, Canu, Necat, and NGSEP), with lengths ranging from 5,991,585 to 6,024,382 bp. In the case of *B. contaminans* TR100, the assembly yielded several contigs (4, 5, 8, and 10) using FLYE, NECAT, CANU, and NGSEP, respectively. In general, the Nanopore-assembled genomes exhibited a low degree of completeness, which improved significantly after polishing using Illumina sequences (Supplementary Figure S3A). We decided to use CANU and FLYE assemblies for downstream analysis because they showed the best completeness and quality characteristics (Supplementary Figure S3B). With these assemblies, we obtained one contig for TP30 and four contigs for TR100, with total sizes of 6,031,870 and 8,476,800 bp, respectively. (Supplementary Figure S3C and S3D). For TR100, we aimed to further investigate whether the different contigs were located on chromosomes or plasmids. Based on a BLAST search, we found that contigs 1, 2, and 3 matched three chromosomes of *B. contaminans* strain CH-1 (ASM472362v1), with coverage of 94%, 88%, and 82%, respectively. The E values were 0.0, and the identities were 99.11%, 99.2%, and 98.78%, respectively. Contig 4 showed partial similarity to four plasmids of *Burkholderia* spp. (*B. contaminans* toggle1 plasmid unnamed3, *B. pseudomallei* strain 2008724860 plasmid p1, *Burkholderia* sp. IDO3 plasmid p1, and *Burkholderia* sp. MBR-1 plasmid pMBR01) with a coverage ranging from 10% to 18% and an identity between 87.51% and 99.94%. The presence of three chromosomes and one plasmid in the TR100 strain was expected, considering that Burkholderia genomes are known for their multi-chromosomal structure, usually consisting of 2-3 chromosomes, and for the additional presence of plasmids (6).

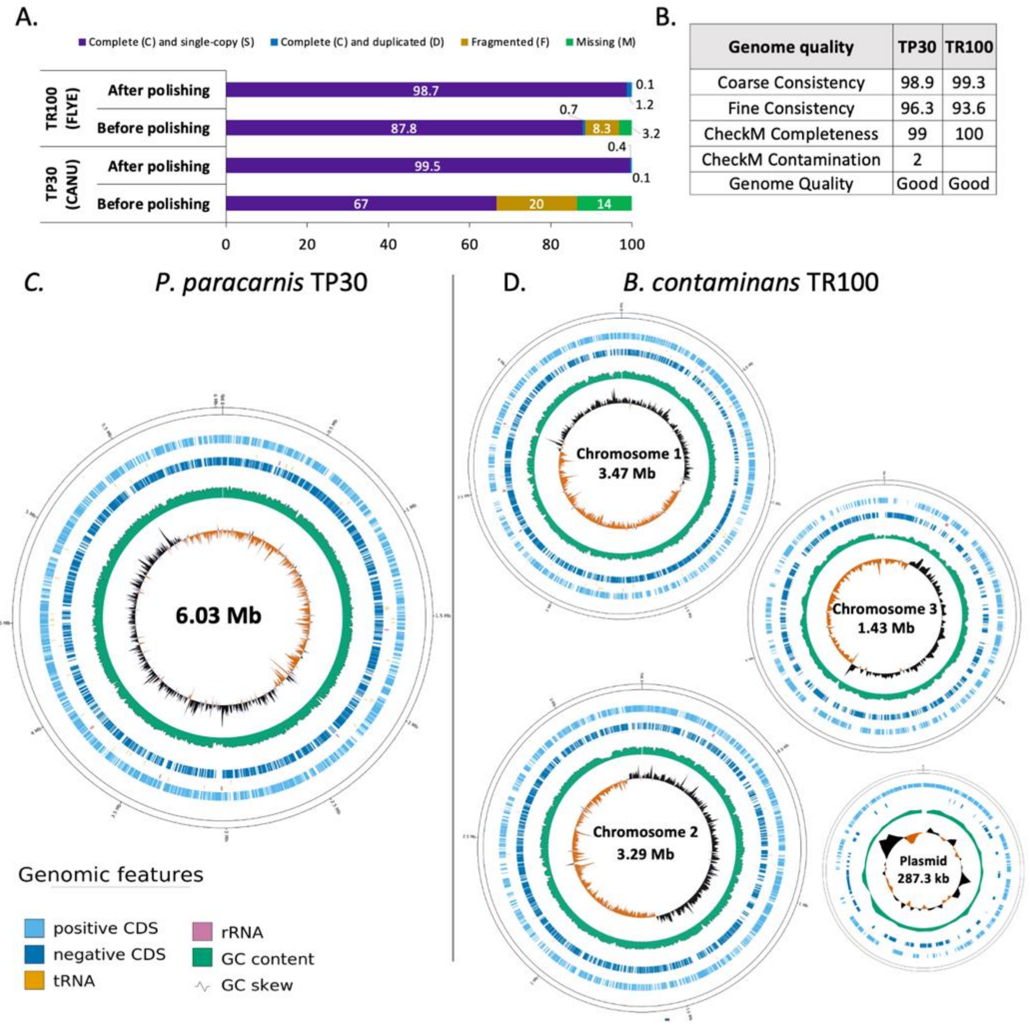

Supplementary Figure S3. A) Genome assembly completeness with BUSCO in *P. paracarnis* TP30 and *B. contaminans* TR100 strains. **B)** Table with genome quality characteristics of TP30 and TR100 genomes. **C)** GenoVi circular map of TP30 genome (one chromosome) and **D)** TR100 genome (3 chromosomes and one plasmid). From outside in, circle one: contig number and length; circle two: positive CDSs; circle three and four: tRNAs and rRNAs; circle five: negative CDSs; circle six: GC content; and circle seven: GC skew.

According to the BLAST-based average nucleotide identity (ANIb) value above 95%, the TP30 genome was classified as *P. paracarnis* (Supplementary Table S5), whereas the TR100 was assigned to *Burkholderia contaminans* (Supplementary Table S6). These results aligned with the phylogenetic analysis based on the multilocus phylogenetic analysis (MLSA) approach (Supplementary Figure S5).

Comparison of the genome-based functional potential of the strains

To compare the overall functional potential of the TP30 and TR100 strains, we annotated their genomes using RAST and Prokka. We were able to assign about 90% and 93% (TP30) and 90% and 98% (TR100) using Prokka and RAST, respectively (Supplementary Table S2). Although Prokka was faster when using multiple annotation tools and databases (e.g., Prodigal, UniProt, and RefSeq) (7), RAST was able to annotate more CDSs (Supplementary Table S2), including those of the *mer* operon. For these reasons, RAST annotations were selected for further analysis, although we use Prokka's annotations for GenoVi Tool analysis and Cluster of Orthologous Genes (COG) classification.

The CDSs distribution of the RAST subsystems was very similar in both strains (Supplementary Figures S4A and S4B). In general, we observed that most of the assigned genes were associated with "amino acids and their derivatives", "carbohydrate", "protein metabolism", and "cofactors, vitamins, prosthetic groups and pigments", which represent about 51% of the assigned genes. However, only 28% and 24% of the TP30 and TR100 CDSs could be assigned to RAST subsystem categories (Supplementary Figures S4A and S4B). Thus, these results only represent a fraction of the total genome information, which likely corresponds to standard functions present in most bacteria and reflects the bias of the databases towards a limited set of well-known proteins from the most commonly cultivable groups (8). Considering this bias, we decided to compare the results with those generated using GenoVi Tool, which is based on Prokka annotation and provides protein classification using the Cluster of Orthologs Group (COG) database.

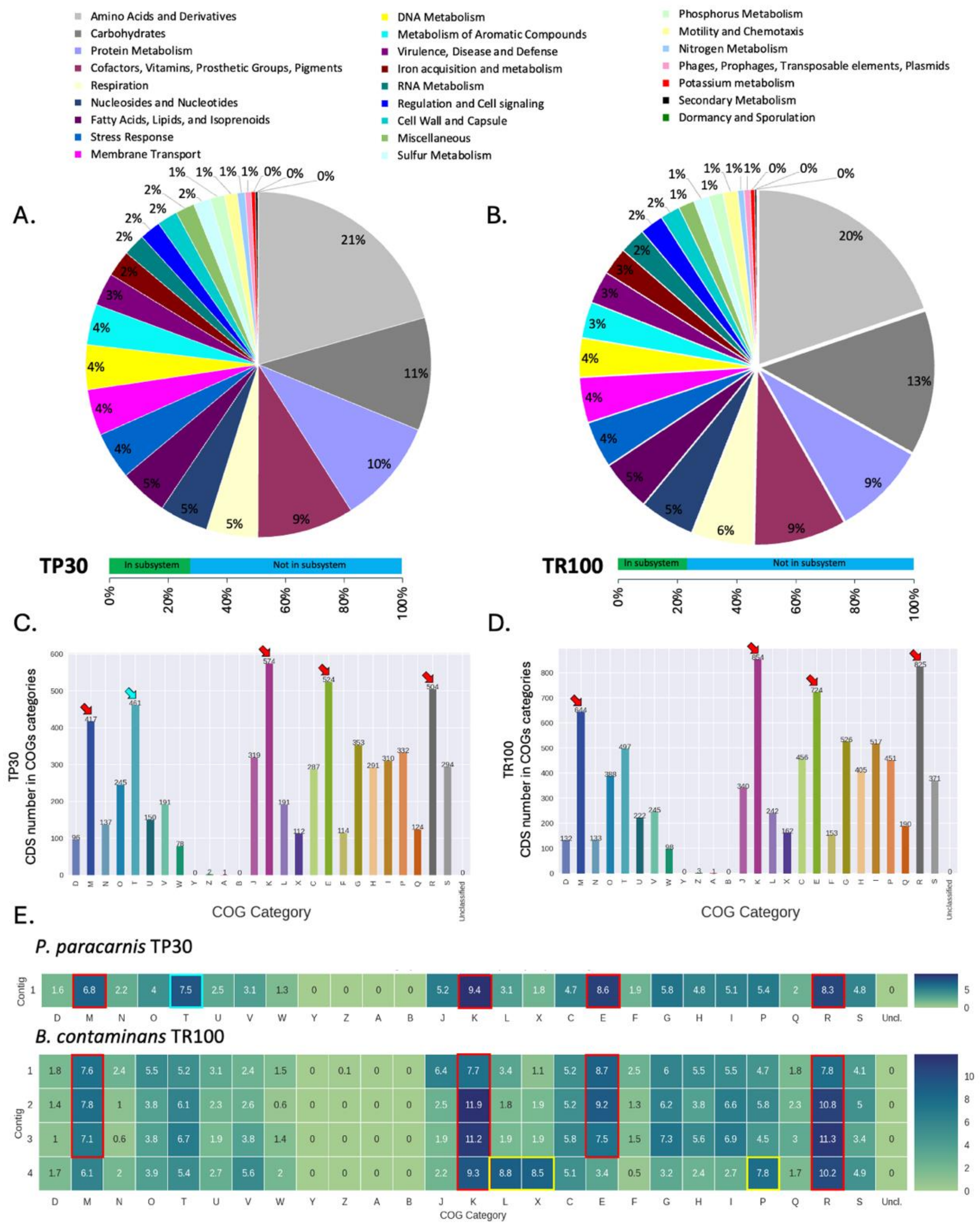

Supplementary Figure S4. Subsystem distribution of **A)** TP30 and **B)** TR100 genomes of RAST annotation server. The percentages of genes that could be assigned to subsystem categories is indicated for each strain as a bar below the distribution: green, present in subsystem categories and blue, absent in the current subsystem categories. CDSs numbers in COGs categories in **C)** TP30 and **D)** TR100 genomes based on PROKKA annotation. **E)** CDSs proportions in in COGs categories: D: Cell cycle control, division, chromosome partitioning; M: Cell wall/membrane/envelope biogenesis; N: Cell

motility; O: Post-translational modification, protein turnover, chaperones; T: Signal transduction mechanism; U: Intracellular trafficking, secretion, and vesicular transport; V: Defense mechanism; W: Extracellular structures; Y: Nuclear structure; Z: Cytoskeleton; A: RNA processing and modification; B: Chromatin structure and dynamics; J: Translation, ribosomal structure, and biogenesis; K: Transcription; L: Replication, recombination, and repair; X: Mobilome: prophages, transposons; C: Energy production and conversion; E: Amino acid transport and metabolism; F: Nucleotide transport and metabolism; G: Carbohydrate transport and metabolism; H: Coenzyme transport and metabolism; I: Lipid transport and metabolism; P: Inorganic ion transport and metabolism; Q: Secondary metabolites biosynthesis, transport, and metabolism; R: General function prediction only and S: Function unknown. The red boxes and arrows indicate the categories of CDSs with the highest frequencies shared between the chromosomes and/or plasmid of both species. The blue box and arrow, and the yellow boxes indicate the category of CDSs with the highest frequencies unique to the *P. paracarnis* TP30 chromosome and *B. contaminans* TR100 potential plasmid, respectively.

The broad COG classification pattern of CDSs in both strains was also similar (Supplementary Figures S4C and S4D), where the major representations of COG categories were "transcription" (K), "amino acid transport and metabolism" (E), "general function prediction" (R), and "cell wall/membrane/envelope biogenesis" (M). Nonetheless, we found a higher number of CDSs in the category "Signal transduction mechanism" (T) for TP30 (Supplementary Figure S4C) and apparent differences between the chromosomes and the plasmid in TR100 (Supplementary Figure S4E). TR100 plasmid was enriched in categories such as "replication, recombination, and repair" (L), "mobilome: prophages, transposons" (X), and "inorganic ion transport and metabolism" (P), which were more represented in the plasmid than in the chromosomes of TP30 and TR100 (Supplementary Figure S4E). On the one hand, the high genetic load devoted to the "transcription" (K) category in both strains and the higher prevalence of "Signal transduction mechanism" (T) CDSs in TP30 are likely related to the need for extensive transcription regulation. The latter is consistent with complex environments and lifestyles, where cells need to respond to frequent variations in environmental conditions (9). On the other hand, the differences found between the plasmidic, and the chromosomic COGs associated with the high recombination frequency (L and X categories) are commonly observed in *Burkholderia* genomes. This attribute is a significant driving force of the diversity and adaptability of this group (10), which may be

related to the presence of Hg and other metal resistance genes in plasmids or ME-like insertion/transposons, phage genes, and plasmids (11, 12).

In addition, the high numbers of CDSs in “general function prediction only” (R) category (Supplementary Figures S4C and S4D) were expected for both strains because it includes proteins with unassigned function, which is consistent with the fact that most of the genes in novel genomes cannot be fully annotated (13). It also agrees with the results generated by RAST annotation, where most of the genes could not be assigned to any RAST category (72% and 76% of the TP30 and TR100 CDSs genomes, respectively). In contrast, the assignment of COGs categories was higher (~85%) (Supplementary Figures S4C and S4D).

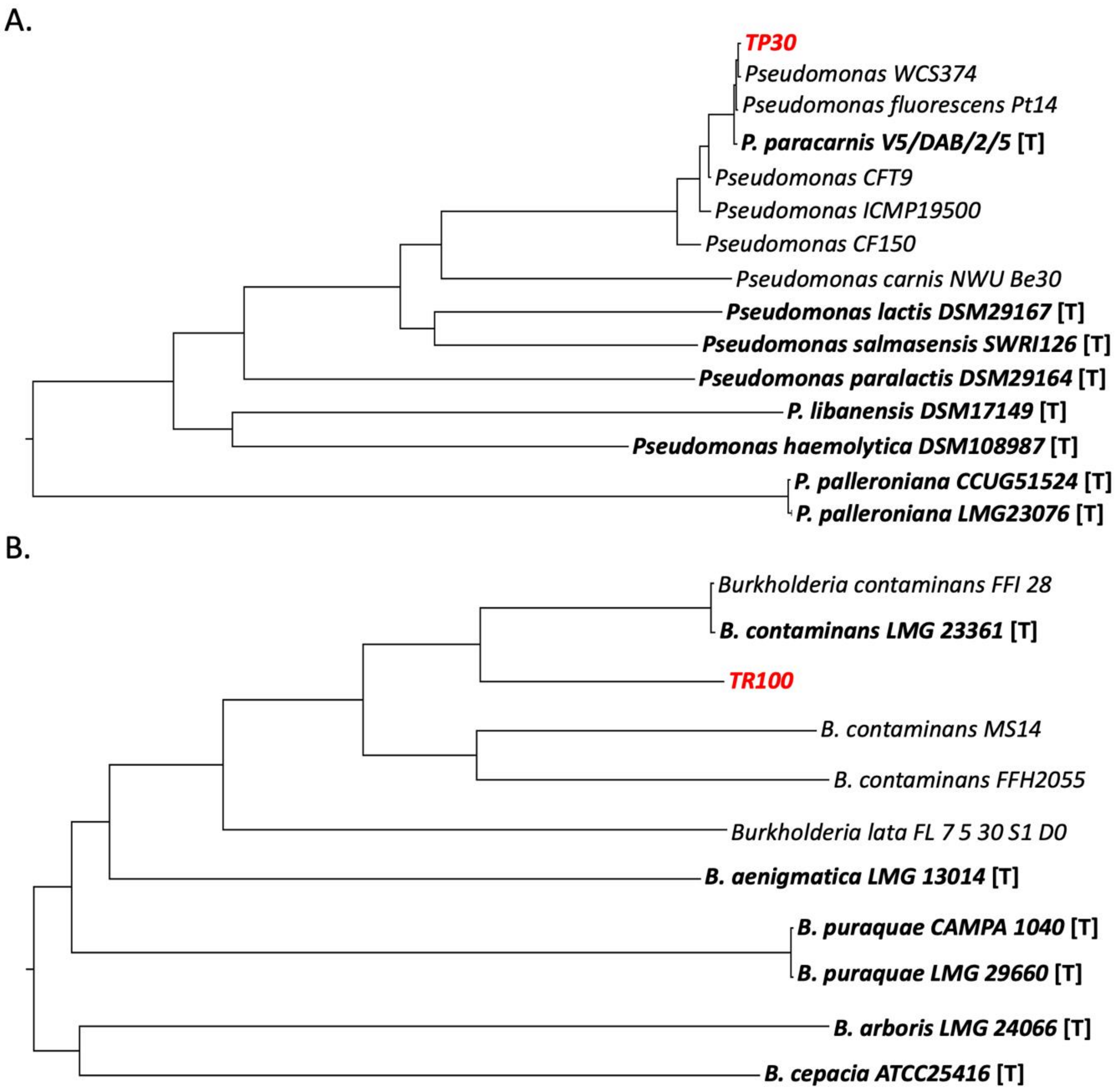

Supplementary Figure S5. Multilocus phylogenetic analysis (MLSA) of **A)** TP30 and **B)** TR100 strains. The phylogenetic reconstruction was based on the concatenation of the sequence of 10 essential genes, as explained in the Methods section.

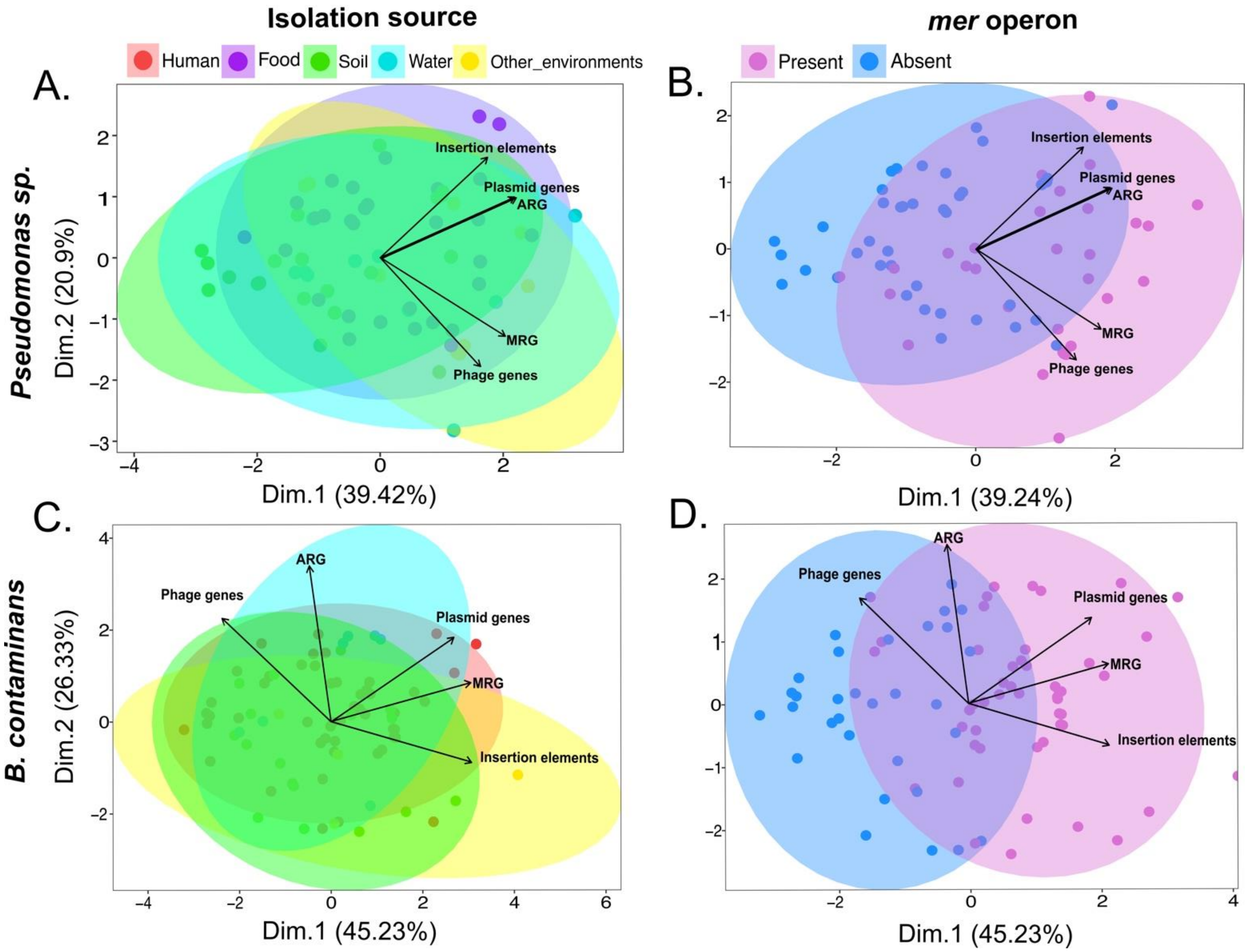

Supplementary Figure S6. Principal Component Analysis (PCA) with the abundance data of MGEs, MRGs, and ARGs genes for *Pseudomonas* spp. **(A-B)** and *B. contaminas* **(C-D)** by isolation source environment **(A and C)** and *mer* operon presence **(B and D)**.

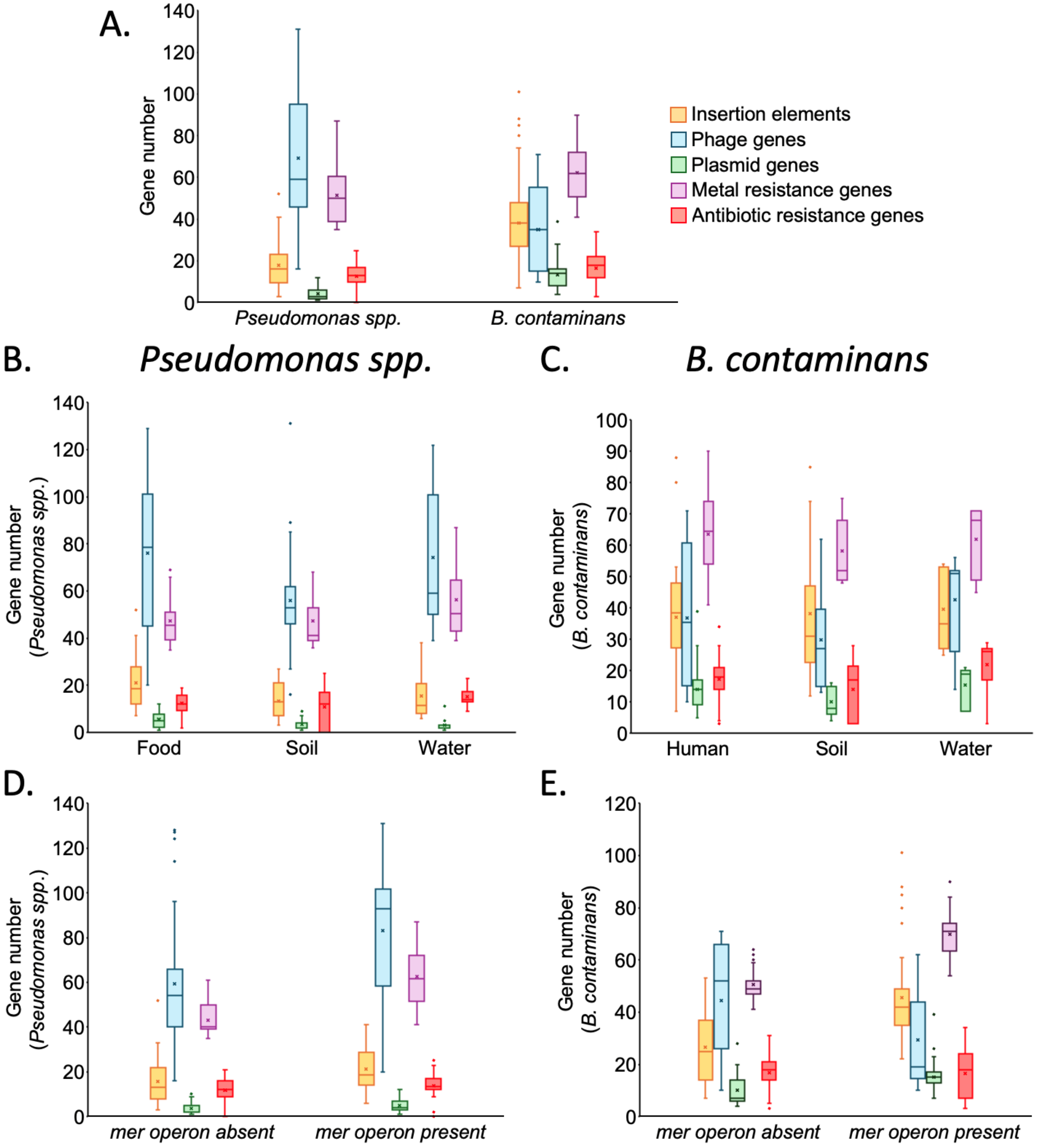

Supplementary Figure S7. Gene number of mobile elements, metal and antibiotic resistance genes by species **(A)**, environmental source of isolation **(B and C)**, and to *mer* operon presence **(D and E)**.

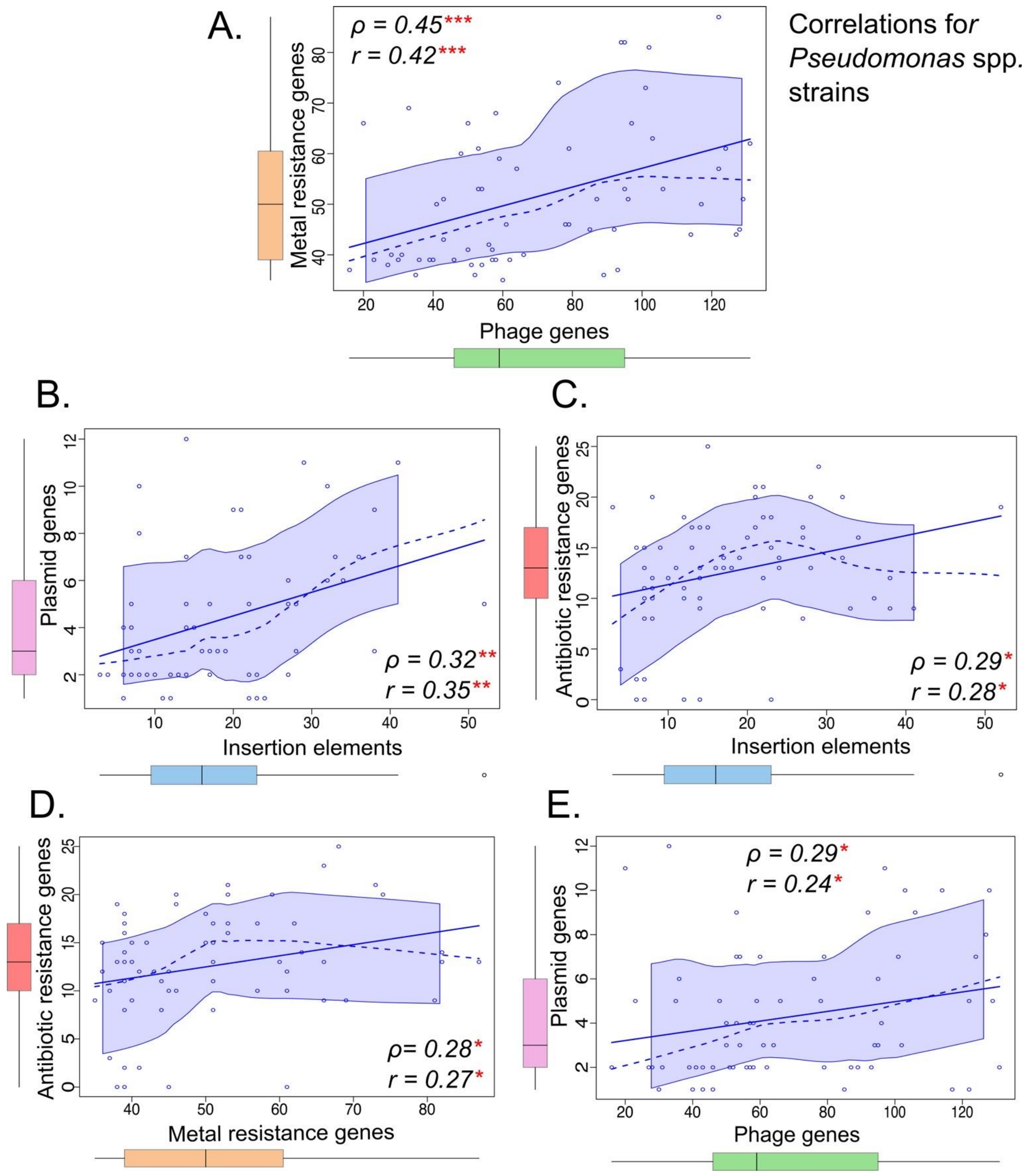

Supplementary Figure S8. Significant Spearman's (ρ) and Pearson's (r) correlations between MGEs, ARGs, and MRGs of *Pseudomonas* spp. (*P. paracarnis, P. carnis* and *P. lactis*) strains.

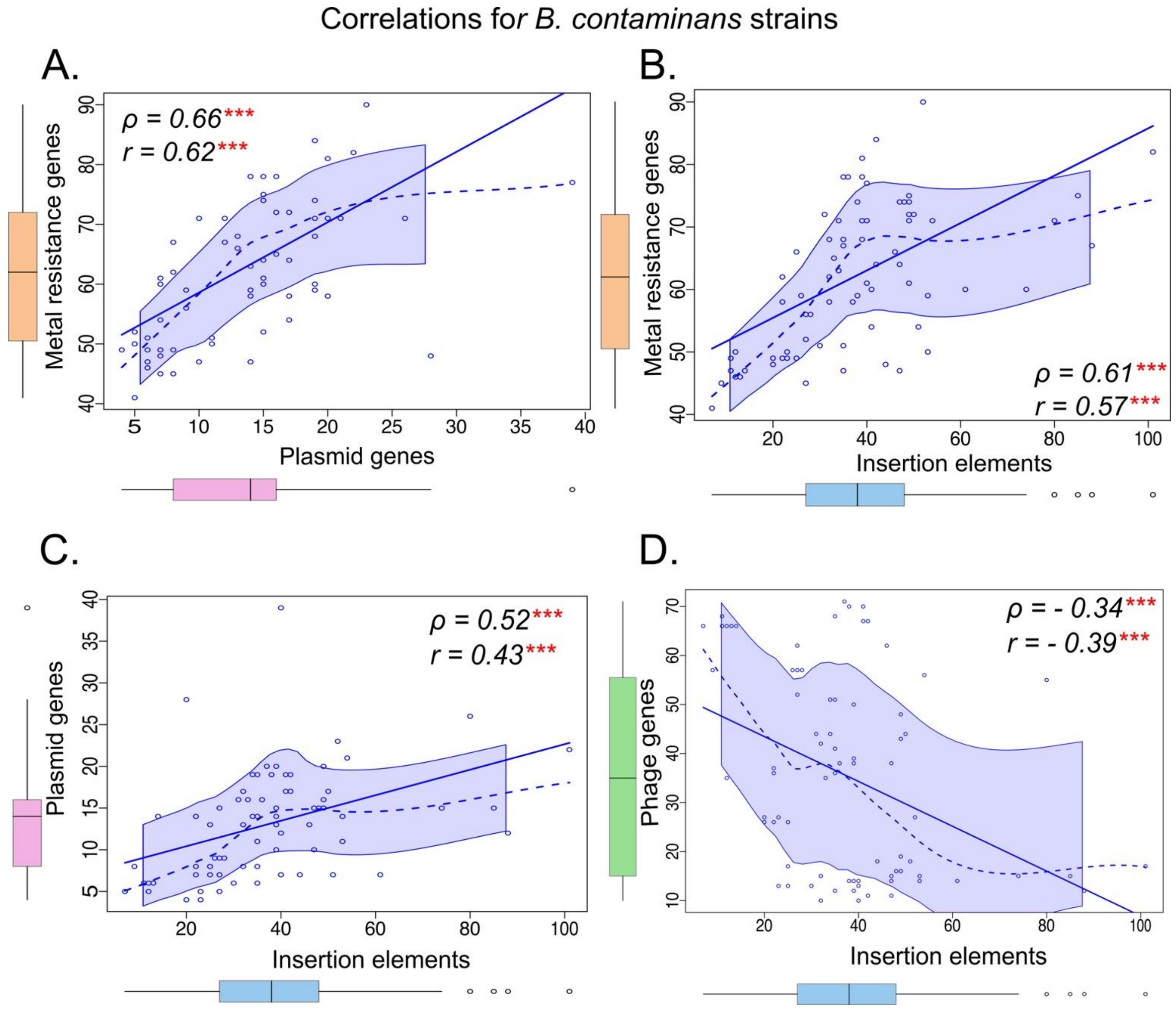

Supplementary Figure S9. Significant Spearman's (ρ) and Pearson's (r) correlations between MGEs, ARGs and MRGs of *B. contaminans* strains.

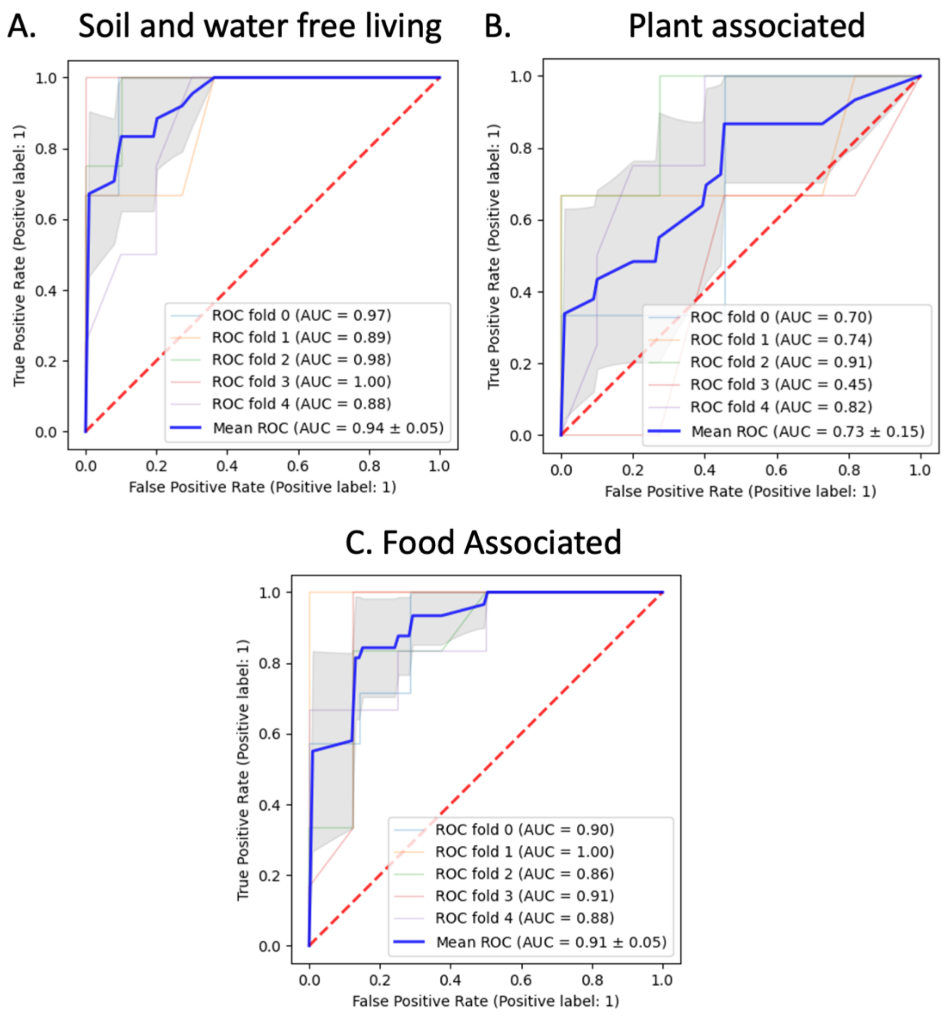

Supplementary Figure S10. Receiver-operating-characteristic (ROC) curves for the *Pseudomonas* lifestyle classifiers. The three panels display five-fold cross-validation results for **(A)** food-associated, **(B)** plant-associated, and **(C)** soil- and water-free-living models. Thin colored lines correspond to individual folds; the thick blue line is the mean ROC, with the mean area under the curve (AUC ± s.d.) indicated in the legend. The red dashed diagonal line indicates random classification. (AUC = 0.5).

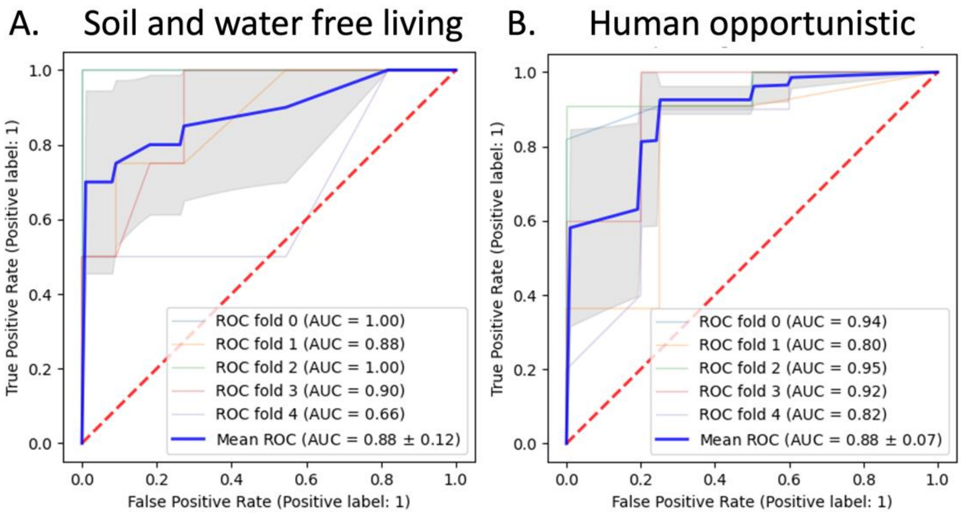

Supplementary Figure S11. Receiver-operating-characteristic (ROC) curves for the *Burkholderia contaminans* lifestyle classifiers. Panels show five-fold cross-validation performance for **(A)** soil and water free-living and **(B)** human-opportunistic models. Thin colored lines correspond to individual folds, while the thick blue line represents the mean ROC; the accompanying legend reports the mean area under the curve (AUC ± s.d.). The red dashed diagonal denotes random classification (AUC = 0.5).

Supplementary Table S1. Illumina and Nanopore sequencing statistics for TP30 and TR100 strains.

	**Raw sequence**	***Pseudomonas paracarnis* TP30**	***Burkholderia contaminans* TR100**
GenBank	BioSample accession no.	SAMN56786084	SAMN56786085
	BioProject accession no.	PRJNA1445321	PRJNA1445321
Illumina	Illumina raw reads	22,408,438	13,436,130
	Q20 (%)		97
	Q30 (%)		92
	GC (%)	60	66
Nanopore	Total bases	301,651,969	350,834,385
	No. of Nanopore reads	26,628	39,602
	No. of Nanopore pass reads	25,197	37,928
	Nanopore read N50 (bp)	23,332	22,183
	Median read quality	10	11
Hybrid		**Assembly**	
	Best assembly	Canu	FLYE
	No. of contigs	1	4
	Chromosome 1 length (bp)	6,031,870	3,474,097
	Chromosome 2 length (bp)		3,287,930
	Chromosome 3 length (bp)		1,427,476
	Plasmid	0	1
	Total potential plasmid length (bp)		287,297
	Total genome size	6,031,870	8,476,800
	GC content (%)	60.17	66.24
	Hybrid assembly N50 (bp)	6,031,870	3,287,930

Supplementary Table S2. Assembly and annotation general results for TP30 and TR100 strains.

Strain	Chromosome/ Plasmid	Size (bp)	%GC	Annotation tool	CDSs	tRNA	rRNA
TP30	1	6,031,870	60.2	RAST	**5,585**	69	19
				Prokka	5,445	**75**	19
TR100	1	3,474,097	66.6	RAST	**3,292**	60	12
				Prokka	3,160	**65**	12
	2	3,287,930	66.5	RAST	**3,162**	5	3
				Prokka	2,893	**11**	3
	3	1,427,476	65.8	RAST	**1,442**	2	3
				Prokka	1,224	**6**	3
	Plasmid	287,297	60.5	RAST	**474**	0	0
				Prokka	367	0	0
	Total	8,476,800	66.24	RAST	**8,360**	67	18
				Prokka	7,644	**82**	18

Supplementary Table S3. Genes mapping and their location in the genome of *P. paracarnis* TP30, by classification within the categories: 1) operon *mer* genes, 2) genes for resistance to other metals, 3) mobile elements, 4) antibiotic resistance genes, and 5) metal tolerance genes (biosurfactants).

Classification	Location	Product
mer operon genes	complement(4949372..4950010)	Organomercurial lyase (EC 4.99.1.2)
	complement(4950023..4951705)	Mercuric ion reductase (EC 1.16.1.1)
	complement(4951741..4952175)	Mercuric transport protein, MerC
	complement(4952188..4952463)	Periplasmic mercury (+2) binding protein, MerP
	complement(4952481..4952831)	Mercuric transport protein, MerT
	4952903..4953334	Mercuric resistance operon regulatory protein MerR
Other metals resistance genes	complement(5841665..5842222)	Chromate reductase (EC 1.6.5.2)
	complement(4465182..4466381)	Chromate transport protein ChrA
	complement(667126..667647)	Copper tolerance protein
	667804..668484	Copper-sensing two-component system response regulator CusR
	668481..669830	Copper sensory histidine kinase CusS
	complement(1158732..1160114)	Multicopper oxidase
	complement(1461275..1462000)	Multicopper polyphenol oxidase
	1546651..1548111	Copper sensory histidine kinase CusS
	complement(1599412..1599609)	Copper(I) chaperone CopZ
	1600204..1602405	Lead, cadmium, zinc and mercury transporting ATPase (EC 3.6.3.3) (EC 3.6.3.5); Copper-translocating P-type ATPase (EC 3.6.3.4)

Classification	Location	Product
	1671796..1672278	Copper metallochaperone PCu(A)C, inserts Cu(I) into cytochrome oxidase subunit II
	1672289..1672690	Putative multicopper oxidases
	complement(2334298..2334849)	Cytochrome oxidase biogenesis protein Cox11-CtaG, copper delivery to Cox1
	2738820..2740943	Lead, cadmium, zinc and mercury transporting ATPase (EC 3.6.3.3) (EC 3.6.3.5); Copper-translocating P-type ATPase (EC 3.6.3.4)
	complement(3180188..3181711)	Apolipoprotein N-acyltransferase / Copper homeostasis protein CutE
	complement(4108865..4109548)	Heavy-metal-associated domain (N-terminus) and membrane-bounded cytochrome biogenesis cycZ-like domain, possible membrane copper tolerance protein
	complement(4109776..4112226)	Type cbb3 cytochrome oxidase biogenesis protein CcoI; Copper-translocating P-type ATPase (EC 3.6.3.4)
	4151806..4153521	Multicopper oxidase
	4153514..4154359	Copper resistance protein B
	4154409..4154795	Copper resistance protein CopC
	4154792..4155673	Copper resistance protein CopD
	complement(4730007..4730867)	Copper resistance protein CopD
	complement(4730871..4731236)	Copper resistance protein CopC
	complement(5091220..5091702)	Copper metallochaperone PCu(A)C, inserts Cu(I) into cytochrome oxidase subunit II
	5377004..5377759	Multicopper polyphenol oxidase
	complement(1548169..1551312)	Cobalt/zinc/cadmium efflux RND transporter, transmembrane protein CzcA
	complement(1551331..1552437)	Cobalt/zinc/cadmium efflux RND transporter, membrane fusion protein CzcB
	complement(1552457..1553683)	Cobalt/zinc/cadmium efflux RND transporter, outer membrane protein CzcC
	complement(1553793..1554698)	Cobalt/zinc/cadmium resistance protein CzcD
	complement(1713874..1714779)	Cobalt-zinc-cadmium resistance protein
	2738820..2740943	Lead, cadmium, zinc and mercury transporting ATPase (EC 3.6.3.3) (EC 3.6.3.5); Copper-translocating P-type ATPase (EC 3.6.3.4)
	1600204..1602405	Lead, cadmium, zinc and mercury transporting ATPase (EC 3.6.3.3) (EC 3.6.3.5); Copper-translocating P-type ATPase (EC 3.6.3.4)
	2432860..2434746	cadmium-translocating P-type ATPase
Mobile elements, plasmids and	5944562..5945914	Mobile element protein
	complement(5963890..5965134)	
	complement(5993382..5994683)	

Classification	Location	Product
phage related genes	complement(264933..265154)	
	complement(265184..265546)	
	complement(265594..266283)	
	complement(1415392..1415808)	
	complement(1488529..1489881)	
	complement(1489960..1491312)	
	1559477..1559869	
	1559893..1560009	
	1560083..1560421	
	1625586..1626938	
	2035218..2036360	
	complement(4200469..4200690)	
	complement(4200720..4201769)	
	4947089..4947433	
	4947471..4948976	
	5175656..5175985	
	5176156..5176602	
	5196422..5197774	
	5593356..5593523	
	2409022..2409819	Chromosome (plasmid) partitioning protein ParA
	2409829..2410701	Chromosome (plasmid) partitioning protein ParB
	complement(3907519..3907899)	Plasmid stabilization system protein
	complement(3948350..3949354)	Phage exonuclease (EC 3.1.11.3)
	complement(4555028..4555771)	Phage exonuclease (ACLAME 70)
	complement(4560456..4561193)	prophage MuSo1, transcriptional regulator, Cro/CI family
	4562089..4562892	Putative DNA-binding protein Roi of bacteriophage BP-933W
	4563661..4564470	Bacteriophage-encoded homolog of DNA replication protein DnaC
	4565765..4566232	Phage NinB DNA recombination
	4566387..4566983	Phage recombination protein NinG
	4566980..4567660	Phage protein
	4571250..4571417	Phage protein
	4571404..4572705	Phage terminase
	4574061..4575173	Phage protein
	4576341..4577312	probable phage protein YPO2110
	4582888..4585845	Phage tail, tail length tape-measure protein H
	4585842..4586180	Phage minor tail protein
	4586177..4586863	Phage tail tip, assembly protein L
	4586871..4587623	Phage tail tip, assembly protein K
	4587623..4588219	Phage tail tip, assembly protein I
	4588229..4592266	Phage tail tip, host specificity protein J
	4594608..4594862	Phage protein

Classification	Location	Product
	4594919..4595344	Phage peptidoglycan hydrolase
	complement(4596920..4597603)	Gifsy-2 prophage protein
	4790944..4791579	Phage repressor protein cI
	complement(4919331..4920395)	Phage integrase
	complement(4920400..4920636)	Phage protein (ACLAME 1394)
	complement(4928740..4929402)	phage-related exonuclease
	complement(4929402..4930391)	Phage exonuclease (ACLAME 70)
	complement(4937765..4938409)	Phage repressor protein cI
	4939546..4940394	Phage antirepressor protein
	4940396..4941373	Phage protein
	4942621..4943199	Phage recombination protein NinG
	4945169..4945498	phage holin, lambda family
	4955423..4956724	Phage terminase
	4960369..4961334	Phage tail, major tail protein V
	4962098..4962529	phage-related conserved hypothetical protein
	4962808..4963176	phage-related conserved hypothetical protein
	4965657..4968728	Phage tail, tail length tape-measure protein H
	4968728..4969066	Phage minor tail protein
	4969076..4969828	Phage tail tip, assembly protein L
	4969831..4970631	Phage tail tip, assembly protein K
	4976712..4977575	Phage antirepressor protein
	4979979..4980200	Phage tail assembly protein I
	4980259..4984260	Phage tail tip, host specificity protein J
	4986606..4986860	Phage protein
	complement(4987979..4988185)	Gifsy-2 prophage protein
	5363460..5364020	Macrophage infectivity potentiator-related protein"
	5555722..5556636	Phage integrase
	520892..521296	Phage holin
	580329..580691	Phage terminase, small subunit
	complement(1023251..1023463)	Phage tail protein GpX
	complement(1030459..1031097)	Phage tail formation protein I
	complement(1031094..1032089)	Phage baseplate assembly protein J
	complement(1324375..1324995)	Gifsy-2 prophage protein
	complement(2001641..2002702)	Phage portal vertex protein GpQ
	complement(2001641..2002702)	Phage portal vertex protein GpQ
	complement(2002702..2004453)	Phage terminase, ATPase subunit GpP
	2004608..2005468	Phage capsid scaffolding protein GpO
	2005500..2006570	Phage major capsid protein GpN
	2006574..2007275	Phage terminase, endonuclease subunit GpM
	2007379..2007843	hypothetical protein
	2007843..2008040	Phage protein
	2008040..2008249	Phage tail protein GpX
	2008340..2008663	Phage holin #Lambda-like group I holin

Classification	Location	Product
	2008660..2009502	Putative phage-encoded peptidoglycan binding protein
	2010287..2010697	Phage tail completion protein GpR
	2010694..2011146	Phage tail completion protein GpS
	2011793..2012146	Phage baseplate assembly protein GpW
	2012143..2013054	Phage baseplate assembly protein GpJ
	2013054..2013668	Phage tail formation protein GpI
	2013665..2015653	Phage tail fiber protein GpH
	2016210..2016347	Phage tail sheath monomer GpFI
	2016503..2017693	Phage tail sheath monomer GpFI
	2017741..2018256	Phage major tail tube protein GpFII
	2018315..2018677	Phage tail fiber protein
	2018686..2018808	Phage P2 GpE family protein
	2018798..2021380	Phage tail length tape-measure protein GpT
	2021387..2021839	Phage tail protein GpU
	2021836..2023113	Phage tail formation protein GpD
	2024984..2025466	Phage protein
	2026509..2029232	Phage protein
	2031812..2032054	Phage protein
	2033884..2034465	Phage protein
	3064893..3066410	putative enzyme; Integration, recombination (Phage or Prophage Related)
	3531429..3532649	Maltoporin (maltose/maltodextrin high-affinity receptor, phage lambda receptor protein)
Antibiotic resistance genes	complement(5844171..5844752)	Streptothricin acetyltransferase, Streptomyces lavendulae type
	complement(771083..771841)	Metal-dependent hydrolases of the beta-lactamase superfamily I
	complement(802188..802604)	Fosfomycin resistance protein FosA
	5677058..5680237	Multidrug efflux system, inner membrane proton/drug antiporter (RND type) => MexF of MexEF-OprN system
	349790..350245	Redox-sensitive transcriptional activator SoxR
	complement(103383..104705)	Uncharacterized MFS-type transporter
Metal tolerance genes (biosurfactants)	5260482..5263829	Trehalose synthase (EC 5.4.99.16)
	complement(5353012..5354757)	Malto-oligosyltrehalose trehalohydrolase (EC 3.2.1.141)
	complement(5348184..5350934)	Malto-oligosyltrehalose synthase (EC 5.4.99.15)

Supplementary Table S4. Genes mapping and their location in the genome of *B. contaminans TR100*, by classification within the categories: 1) operon *mer* genes, 2) genes for resistance to other metals, 3) mobile elements, 4) antibiotic resistance genes, and 5) metal tolerance genes (biosurfactants).

	Classification	Location	Product
Chromosome 1	Other metals resistance genes	complement (65373..66539)	Chromate transport protein ChrA
		complement(242588..243859)	Chromate transport protein ChrA
		1895868..1896479	Chromate transport protein ChrA
		1896476..1897015	Chromate transport protein ChrA
		3001603..3002241	Chromate transport protein ChrA
		3002238..3002765	Chromate transport protein ChrA
		126032..126406	Copper resistance protein CopC
		880786..881625	Multicopper polyphenol oxidase
		complement(1610315..1611964)	Multicopper oxidase
		complement(1612032..1613477)	Multicopper oxidase
		1667499..1667945	Copper metallochaperone PCu(A)C, inserts Cu(I) into cytochrome oxidase subunit II
		complement(1968262..1968693)	Heme/copper-type cytochrome/quinol oxidases, subunit 2
		complement(2671335..2673962)	Lead, cadmium, zinc and mercury transporting ATPase (EC 3.6.3.3) (EC 3.6.3.5); Copper-translocating P-type ATPase (EC 3.6.3.4)
		complement(3025319..3025519)	Copper(I) chaperone CopZ
		3025868..3026200	Heme/copper-type cytochrome/quinol oxidases, subunit 2
		3076387..3077112	Cytoplasmic copper homeostasis protein CutC
		3179812..3180420	Cytochrome oxidase biogenesis protein Cox11-CtaG, copper delivery to Cox1
		complement(3357584..3359272)	Apolipoprotein N-acyltransferase / Copper homeostasis protein CutE
		3383969..3384901	Copper resistance protein CopD
		complement(2563113..2564270)	Cobalt/zinc/cadmium resistance protein CzcD
		complement(576942..580160)	CzcABC family efflux RND transporter, transmembrane protein
	Mobile genetic elements, plasmids and Phage related genes	1268822..1269223	Mobile element protein
		1690863..1691915	
		3418533..3418781	
		complement(888432..888614)	
		complement(1241199..1241609)	
		complement(2707868..2708761)	Chromosome (plasmid) partitioning protein ParB
		complement(2708793..2709572)	Chromosome (plasmid) partitioning protein ParA
		1174036..1174659	Plasmid conjugative transfer endonuclease
		complement(256167..256541)	Phage protein
		1544642..1545043	Phage protein
		2020739..2021128	Phage holin
	Antibiotic resistance genes	complement(2315501..2316712)	Metallo-beta-lactamase superfamily protein PA0057
		15466..16662	Beta-lactamase class C-like and penicillin binding proteins (PBPs) superfamily
		541007..541783	Metal-dependent hydrolases of the beta-lactamase superfamily I
		complement(1117913..1119112)	Multidrug efflux system, membrane fusion component => MexX of of MexXY/AxyXY

	Classification	Location	Product
	Metal tolerance genes (biosurfactants)	complement(1665816..1666568)	Trehalose-6-phosphate phosphatase (EC 3.1.3.12)
Chromosome 2	Other metals resistance genes	complement(70518..73370)	Lead, cadmium, zinc and mercury transporting ATPase (EC 3.6.3.3) (EC 3.6.3.5); Copper-translocating P-type ATPase (EC 3.6.3.4)
		3185512..3186066	Chromate reductase (EC 1.6.5.2)
		complement(661100..661546)	Copper metallochaperone PCu(A)C, inserts Cu(I) into cytochrome oxidase subunit II
		complement(661627..661992)	Copper resistance protein CopC
		complement(960519..960851)	Copper binding protein, plastocyanin/azurin family
		complement(2452497..2453927)	Copper sensory histidine kinase CusS
		complement(2453924..2454607)	Copper-sensing two-component system response regulator CusR
		complement(2740788..2741153)	Copper resistance protein CopC
		complement(2806942..2810148)	Copper/silver efflux RND transporter, transmembrane protein CusA
		complement(2810145..2811686)	Copper/silver efflux RND transporter, membrane fusion protein CusB
		complement(2811698..2812834)	Copper/silver efflux RND transporter, outer membrane protein CusC
		1556243..1556908	Cobalt/zinc/cadmium resistance protein CzcD
		complement(2454607..2457819)	Cobalt/zinc/cadmium efflux RND transporter, transmembrane protein CzcA
		complement(2457945..2459405)	Cobalt/zinc/cadmium efflux RND transporter, membrane fusion protein CzcB"
		complement(2459440..2460753)	Cobalt/zinc/cadmium efflux RND transporter, outer membrane protein CzcC
		complement(2800843..2802138)	Cobalt/zinc/cadmium resistance protein CzcD
	Mobile genetic elements, plasmids and phage related genes	complement(2239881..2240822)	Mobile element protein
		complement(2758367..2758528)	
		complement(3019207..3020316)	
		3179584..3180951	Protein involved in initiation of plasmid replication
		complement(3181042..3182103)	Chromosome (plasmid) partitioning protein ParB
		1404813..1405229	plasmid stability protein
		complement(1819369..1819728)	Phage protein
		complement(2022671..2023213)	Phage endolysin
		complement(2292400..2293695)	
		2599595..2600152	Macrophage infectivity potentiator-related protein
		complement(3068648..3070489)	Phage T7 exclusion protein
		3079093..3079536	Phage integrase
		3079562..3079717	Phage integrase
		complement(677139..678080)	Abortive infection bacteriophage resistance protein
		complement(1016991..1018028)	Bacteriophage protein gp37
		complement(1029999..1031198)	Phage recombination protein Bet
		1046053..1047534	Phage terminase, large subunit
		1049018..1049773	Phage protein YqbB
		1053442..1053825	Phage protein
		1053854..1054333	
		1054439..1054765	
		1054770..1055360	
		1055370..1057181	

	Classification	Location	Product
		1057196..1057636	
		1057639..1058211	
		1058395..1060407	
		1060404..1060979	
		1060979..1061296	probable bacteriophage protein STY1063
		1061293..1062261	Phage protein
		1062753..1063379	
		1063387..1063740	
		1063737..1064918	
		1064918..1065580	
		1068902..1069399	Phage lysozyme R (EC 3.2.1.17)
		1070333..1070812	Homology to phage-tail assembly proteins
	Antibiotic resistance genes	2844290..2844880	Streptothricin acetyltransferase, Streptomyces lavendulae type
		3270334..3271182	Protein involved in biosynthesis of mitomycin antibiotics/polyketide fumonisin
		237570..240770	RND efflux system, inner membrane transporter
Chromosome 3	Other metals resistance genes	1161895..1162806	Chromate resistance protein ChrB
		1162803..1163981	Chromate transport protein ChrA
	Mobile genetic elements, plasmids and phage related genes	610831..612030	Mobile element protein
		1407395..1407748	
		1407779..1409374	
		complement(2747..2935)	
		46063..46215	
		46332..46571	
		523996..524139	
		90338..91033	Chromosome (plasmid) partitioning protein ParA
		91026..92057	Chromosome (plasmid) partitioning protein ParB
		complement(92153..93469)	Protein involved in initiation of plasmid replication
	Antibiotic resistance genes	complement(1282268..1283437)	Class C beta-lactamase (EC 3.5.2.6)
		complement(524463..525338)	Protein involved in biosynthesis of mitomycin antibiotics/polyketide fumonisin
		518293..519171	Protein involved in biosynthesis of mitomycin antibiotics/polyketide fumonisin
	Metal tolerance genes (biosurfactants)	complement(765616..767487)	Malto-oligosyltrehalose trehalohydrolase (EC 3.2.1.141)
		complement(760629..763403)	Malto-oligosyltrehalose synthase (EC 5.4.99.15)
		complement(450418..451821)	Metallo-beta-lactamase family protein,RNA-specific
Plasmid	*mer* operon genes	178116..178235	Mercuric transport protein, MerC
		complement(179314..179574)	Mercuric resistance operon regulatory protein MerR
		179804..180169	Mercuric transport protein, MerT
		180182..180457	Periplasmic mercury(+2) binding protein, MerP
		180557..180907	Mercuric transport protein, MerC
		180947..181249	Mercuric ion reductase (EC 1.16.1.1)
		181270..182625	Mercuric ion reductase (EC 1.16.1.1)
		182694..183458	Organomercurial lyase (EC 4.99.1.2)
		183695..183928	Mercuric transport protein, MerE
	Other metals resistance genes	complement(205760..208966)	Copper/silver efflux RND transporter, transmembrane protein CusA
		complement(208963..210492)	Copper/silver efflux RND transporter, membrane fusion protein CusB

	Classification	Location	Product
		complement(210504..211796)	Copper/silver efflux RND transporter, outer membrane protein CusC
		154875..156674	Copper tolerance protein
		156807..157970	Multicopper oxidase
		158112..158384	RND efflux system, membrane fusion protein
		158448..158822	Copper resistance protein CopC
		158822..159763	Copper resistance protein CopD
		complement(160754..161971)	Multicopper oxidase
		complement(161968..162297)	Multicopper oxidase
		complement(162370..164826)	Lead, cadmium, zinc and mercury transporting ATPase (EC 3.6.3.3) (EC 3.6.3.5); Copper-translocating P-type ATPase (EC 3.6.3.4)
		complement(165062..165259)	Copper chaperone
		complement(162370..164826)	Lead, cadmium, zinc and mercury transporting ATPase (EC 3.6.3.3) (EC 3.6.3.5); Copper-translocating P-type ATPase (EC 3.6.3.4)
		170468..170923	Cobalt-zinc-cadmium resistance protein
	Mobile elements, plasmids and phage related genes	188199..188582	Mobile element protein
		complement(189597..191102)	Mobile element protein
		complement(191096..192529)	
		complement(212976..215972)	
		complement(131400..134381)	
		complement(138612..138785)	
		168942..170108	
		276341..276937	IncF plasmid conjugative transfer pilus assembly protein TraB
		280547..281620	IncF plasmid conjugative transfer pilus assembly protein TraC
		283377..283955	IncF plasmid conjugative transfer pilus assembly protein TraW
		283967..285022	IncF plasmid conjugative transfer pilus assembly protein TraU
		complement(45238..45489)	Phage protein (ACLAME 728)
		complement(45592..46182)	Phage protein
		57953..58858	Bacteriophage protein gp37
		82820..83320	Phage protein
		118722..119294	Phage hydrolase of HD superfamily (COG1896) (ACLAME 764)

Supplementary Table S5 (Excel file). ANIb obtained from the comparison of the genome of strain TP30 with type strains (*P. carnis* [T], *P. paracarnis* V5/DAB/2/5 [T], *P. lactis* DSM 29167 [T], and *P. paralactis* DSM 29164 [T]) and 70 genomes of *Pseudomonas* spp. ANIb above 95% can indicate that two genomes belong to the same species. Colored numbers indicate misclassified strains of: *1) *P. paracarnis*, *2) *P. lactis*, and *3) *P. carnis*.

Supplementary Table S6 (Excel file). ANIb obtained from the comparison of the genome of strain TR100 with type strain *B. contaminans* LMG 23361 [T] and 79 genomes of *B. contaminans*. ANIb above 95% may indicate that two genomes belong to the same species. Red number *1 indicate misclassified strains: *B. contaminans* AuBur16, and GalA64

Supplementary Table S7. General information of the genomes selected as closely related strains to *P. paracarnis TP30.*

Genome	Contigs	Genes	Completeness	Contamination	Fragmentation	Number of *mer*A genes	Number of *mer*B genes	Isolation source
Pseudomonas carnis 200188 6	146	5033	95.17	0.05	1.2	0	0	Food
Pseudomonas carnis B4 2	85	5522	99.75	0.31	0.84	0	0	Food
Pseudomonas carnis Bi08	117	5989	99.6	0.15	0.69	0	0	Soil
Pseudomonas carnis Bi110	62	5982	99.67	0.15	0.46	0	0	Soil
Pseudomonas carnis Bi111	169	5976	99.52	0.15	0.65	0	0	Soil
Pseudomonas carnis Bi64	127	6009	99.72	0.18	0.52	0	0	Soil
Pseudomonas carnis MF7446	44	5465	99.65	0.15	0.54	0	0	Food
Pseudomonas carnis MF7452	73	5769	99.29	0.28	0.89	0	0	Food
Pseudomonas carnis MF7454	78	6197	99.67	0.23	0.8	1	0	Food
Pseudomonas carnis PSM2	90	5054	96.13	0.18	0.92	0	0	Food
Pseudomonas carnis UCD MED7	132	5558	99.57	0.15	0.51	0	0	Food
Pseudomonas carnis UMB248	57	4866	91.25	0.18	0.85	0	0	Food
Pseudomonas lactis DSM 29142	60	6066	99.72	0.49	0.97	1	1	Food
Pseudomonas lactis DSM 29167	42	6016	99.49	0.23	0.74	0	0	Food
Pseudomonas lactis FSL E2 0548	244	6221	99.47	4.5	1.16	1	0	Food
Pseudomonas lactis FSL E2 8864	188	6033	99.22	0.51	1.68	0	0	Food
Pseudomonas lactis FSL R10 2514	57	5661	99.27	0.15	0.71	0	0	Food
Pseudomonas lactis FSL R5 0199	97	6052	99.72	0.23	0.97	3	2	Food
Pseudomonas lactis FSL W5 0203	113	5657	99.7	0.2	0.74	0	0	Food
Pseudomonas lactis ITEM 1729	65	5634	99.7	0.2	0.59	0	0	Food
Pseudomonas lactis ITEM 17299	137	5626	99.6	0.28	0.79	0	0	Food
Pseudomonas lactis MF7445	102	6045	99.34	0.33	1.1	1	0	Food
Pseudomonas lactis SS101	1	5376	99.44	0.13	0.51	0	0	Soil
Pseudomonas lactis PA4C2	5	5402	99.27	0.08	0.61	1	0	Soil
Pseudomonas lactis UKR1	1139	5684	95.96	0.77	2.98	1	0	Soil
Pseudomonas lactis WS 4997	28	5943	99.47	0.23	0.79	1	0	Food
Pseudomonas lactis WS 5000	26	5850	99.49	0.26	0.83	0	0	Food
Pseudomonas lactis WS 5404	46	6119	99.49	2.1	0.81	0	0	Food
Pseudomonas lactis WS 5405	54	6099	99.47	0.23	0.75	0	0	Food
Pseudomonas paracarnis 1923	47	5648	99.9	0.05	0.59	2	1	Water
Pseudomonas paracarnis A006	99	5911	99.8	0.2	0.94	1	0	Food
Pseudomonas paracarnis b191	84	5655	99.22	0.31	0.84	1	1	Other environments
Pseudomonas paracarnis HFA27 1	134	5848	99.82	0.23	0.64	1	0	Other environments
Pseudomonas paracarnis PS01857	282	5841	99.85	0.13	0.86	2	1	Other environments
Pseudomonas paracarnis RQ057	52	5535	99.8	0.15	0.78	0	0	Food

Genome	Contigs	Genes	Completeness	Contamination	Fragmentation	Number of *mer*A genes	Number of *mer*B genes	Isolation source
Pseudomonas paracarnis UBT403	4	5320	99.75	0.05	0.46	0	0	Food
Pseudomonas paracarnis V5 DAB 2 5	127	5501	99.85	0.41	0.82	0	0	Food
Pseudomonas paralactis DSM 29164	37	5302	98	0.13	0.82	0	0	Food
Pseudomonas paralactis SWRI70	123	5310	98.03	0.1	0.83	0	0	Soil
Pseudomonas sp AP42	227	6087	98.96	0.05	0.89	1	0	Water
Pseudomonas sp CF150	95	5457	97.62	0.26	0.98	1	0	Soil
Pseudomonas sp CFT9	67	5623	97.9	0.23	0.94	2	1	Water
Pseudomonas sp FP1911	1	5321	99.85	0.08	0.4	0	0	Soil
Pseudomonas sp FW305 124	177	5192	99.75	0.08	0.46	0	0	Water
Pseudomonas sp GBPI 506	75	5917	99.65	0.15	0.65	1	1	Soil
Pseudomonas sp HMWF011	344	5525	97.02	1.15	5.1	0	0	Water
Pseudomonas sp HMWF034	107	5519	99.14	0.51	1.94	0	0	Water
Pseudomonas sp ICMP 19500	144	5850	99.7	0.15	1.07	1	0	Other environments
Pseudomonas sp PS887	88	5400	99.85	0.15	0.56	0	0	Soil
Pseudomonas sp SWRI22	3	5189	99.75	0.18	0.45	0	0	Soil
Pseudomonas sp WCS374	1	5406	99.85	0.2	0.47	2	2	Soil
Pseudomonas paracarnis TP30	1	5421	99.85	0.49	0.83	1	1	Soil
uncultured proteobacterium GCA 903871155.1	173	5648	99.72	0.26	0.71	1	0	Water
uncultured Pseudomonas sp GCF 017992845.1	73	5379	96.56	0.2	0.84	1	1	Water
Pseudomonas carnis B157	68	5916	99.82	0.22	0.81	1	0	Food
Pseudomonas carnis DR54	135	5384	98.84	0.09	0.79	0	0	Soil
Pseudomonas carnis LP	86	5891	99.82	0.2	0.58	1	1	Food
Pseudomonas carnis MED3	146	5561	99.6	0.18	0.51	0	0	Food
Pseudomonas fluorescens A506	2	5391	99.91	0.36	0.81	0	0	Soil
Pseudomonas fluorescens FH5	87	5512	99.91	0.02	0.71	1	0	Water
Pseudomonas fluorescens Pt14	1	5192	99.89	0.22	0.58	0	0	Soil
Pseudomonas lactis ERR7163067 maxbin2 33	375	5466	96.51	0.36	1.81	1	0	Water
Pseudomonas lactis IHBB15160	52	5520	99.42	0.13	0.82	0	0	Soil
Pseudomonas lactis PpR24	119	5431	99.47	0.04	0.59	0	0	Soil
Pseudomonas lactis RS3R 2	85	5425	99.51	0.2	0.71	0	0	Soil
Pseudomonas paracarnis USMM045	142	5673	99.89	0.11	0.56	2	1	Other environments
Pseudomonas paracarnis USMM046	142	5671	99.89	0.11	0.5	2	1	Other environments
Pseudomonas paracarnis USMM047	134	5672	99.89	0.11	0.56	2	1	Other environments
Pseudomonas paracarnis USMM048	139	5672	99.89	0.11	0.5	2	1	Other environments
Pseudomonas paracarnis Y128	88	5308	99.82	0.07	0.51	0	0	Other environments
Pseudomonas paracarnis Y21	81	5304	99.8	0.07	0.47	0	0	Other environments

Supplementary Table S8. General information of the genomes selected as closely related strains to *B.contaminans TR100.*

Genome	Contigs	Genes	Completeness	Contamination	Fragmentation	Number of *mer*A genes	Number of *mer*B genes	Isolation source
B. contaminans 170816	6	7808	97.43	0.38	1.46	**3**	**3**	Human
B. contaminans 172630038 1	6	7721	100	0.11	0.4	1	1	Water
B. contaminans 40H	102	7616	99.92	0.04	0.6	2	1	Human
B. contaminans 5080	6	7798	100	0.15	0.59	0	0	Human
B. contaminans 51242556	74	8072	99.98	0.04	0.42	1	1	Human
B. contaminans 51242837	70	8073	99.98	0.04	0.38	1	1	Human
B. contaminans 51243042	75	8069	99.98	0.04	0.38	1	1	Human
B. contaminans 51243098	102	8075	99.98	0.04	0.52	1	1	Human
B. contaminans 51276351	82	8070	99.98	0.04	0.44	1	1	Human
B. contaminans 584U	96	7285	99.92	0.06	0.82	0	0	Human
B. contaminans 661U	121	7294	99.91	0.06	0.87	0	0	Human
B. contaminans AU21922	94	7656	99.92	0.13	0.73	0	0	Human
B. contaminans AU31268	548	8091	98.99	0.21	2.53	1	1	Human
B. contaminans AU33395	89	7880	99.98	0.06	0.69	**3**	**3**	Human
B. contaminans AU34969	67	7832	99.98	0.04	0.38	1	1	Human
B. contaminans AU36501	76	7446	98.29	0.1	1.13	1	1	Human
B. contaminans AU41584	46	7531	98.01	0.11	0.93	1	1	Human
B. contaminans AU41716	149	8597	99.38	0.42	1.28	**2**	**2**	Human
B. contaminans AU44216	85	7826	99.98	0.04	0.47	1	1	Human
B. contaminans AU44977	81	6543	91.8	0.06	0.59	0	0	Human
B. contaminans AU44979	116	8039	99.77	0.11	0.88	**2**	**0**	Human
B. contaminans AU7143	133	7919	99.92	0.04	0.74	1	1	Human
B. contaminans AuBur16	2744	7796	94.58	0.19	0.7	0	0	Soil
B. contaminans B17 01563 1	4	7556	99.96	0.08	0.32	1	1	Other_environments
B. contaminans BCC0123	41	7818	100	0.04	0.53	**3**	**3**	Human
B. contaminans BCC0254	51	7396	99.31	0.11	0.85	0	0	Human
B. contaminans Bp8999	108	7391	99.47	0.11	1.27	0	0	Soil
B. contaminans Bp9000	74	7127	99.62	0.08	1.06	0	0	Soil
B. contaminans Bp9001	79	7544	99.72	0.19	1.18	0	0	Soil
B. contaminans Bp9018	86	7374	99.27	0.17	0.9	0	0	Water
B. contaminans Bp9025	95	7453	97.58	0.23	1.28	0	0	Water
B. contaminans CAMPA 1773	68	7614	99.98	0.06	0.45	1	1	Human
B. contaminans CAMPA 1809	106	7987	99.98	0.06	0.8	**3**	**3**	Human

Genome	Contigs	Genes	Completeness	Contamination	Fragmentation	Number of *mer*A genes	Number of *mer*B genes	Isolation source
B. contaminans CAMPA 187	127	7274	99.94	0.13	0.97	0	0	Human
B. contaminans CAMPA 188	46	6119	93.74	0.11	0.63	0	0	Human
B. contaminans CAMPA 315	38	7237	99.94	0.08	0.74	0	0	Human
B. contaminans CAMPA 320	87	7938	99.98	0.04	0.65	**3**	**3**	Human
B. contaminans CAMPA 514	44	7876	99.92	10.95	0.85	0	0	Human
B. contaminans CAMPA 688	60	7409	99.94	0.08	0.8	0	0	Human
B. contaminans CCUG59607	162	7104	98.37	0.4	2.14	1	1	Human
B. contaminans DM32	4	7678	97.99	0.13	0.88	1	1	Other_environments
B. contaminans FFH2055	8	7248	99.87	0.21	0.81	0	0	Human
B. contaminans FFI 28	239	7470	98.86	0.11	1.71	0	0	Other_environments
B. contaminans FL 1 2 30 S1 D0	3	7143	99.34	0.11	0.75	0	0	Soil
B. contaminans GalA64	3	6739	89.08	0.23	0.66	0	0	Soil
B. contaminans HI4235	73	8006	100	0.04	0.66	1	1	Soil
B. contaminans ID 252 23	360	7588	97.79	0.29	2.83	1	0	Human
B. contaminans ID 253 23	223	7583	98.82	0.19	1.87	1	0	Human
B. contaminans ID 254 23	162	7344	98.91	0.27	1.57	0	0	Human
B. contaminans ID 256 23	185	7337	98.52	0.19	1.48	0	0	Human
B. contaminans ID 291 23	266	7641	98.05	0.17	2.17	1	0	Human
B. contaminans ID 617 23	205	7628	99.19	0.17	1.39	0	0	Human
B. contaminans ID 619 23	214	7611	99.1	0.19	1.81	0	0	Human
B. contaminans ID 645 23	232	7805	99.01	0.25	1.83	0	0	Human
B. contaminans LMG 16227	101	7392	99.74	0.27	1.5	1	1	Human
B. contaminans LMG 23253	101	7715	99.96	0.04	0.7	**2**	**1**	Human
B. contaminans LMG 23255	80	6650	93.85	0.02	0.46	**2**	**1**	Human
B. contaminans LMG23361	7	8572	99.57	0.61	1.53	1	1	Other_environments
B. contaminans MS14	3	7516	99.61	2.01	0.97	0	0	Soil
B. contaminans NML 151013	5	7741	100	0.06	0.74	1	1	Human
B. contaminans NML151067	5	7733	99.98	0.04	0.61	1	1	Human
B. contaminans OYA 0603	141	7703	99.76	0.04	1.22	1	1	Soil
B. contaminans p14	68	7617	100	0.06	0.51	0	0	Soil
B. contaminans PB AQ24	169	8037	99.61	0.04	0.76	1	1	Soil
B. contaminans R 18442	68	7473	99.76	0.19	1.18	0	0	Human
B. contaminans R 37747	163	8039	99.92	0.1	1.1	2	1	Human
B. contaminans R 71171	47	7255	97.71	0.21	0.78	0	0	Human
B. contaminans SBC01	19	7925	99.76	0.04	0.49	1	1	Human
B. contaminans SCAID TST1 2021	6	7481	95.65	1.11	2.34	0	0	Human
B. contaminans toggle1	6	8343	99.83	0.38	2.2	1	1	Human

Genome	Contigs	Genes	Completeness	Contamination	Fragmentation	Number of *mer*A genes	Number of *mer*B genes	Isolation source
B. contaminans TR100	4	7601	99.17	0.25	1	1	1	Soil
B. contaminans XL73	5	7647	99.96	0.04	0.41	1	1	Soil
B. contaminans ZCC	5	8061	99.66	0.8	2.1	1	1	Soil
B. contaminans ZCC50	197	7932	99.94	0.04	0.69	1	1	Soil
Burkholderia sp. 4M9327G5	259	8280	99.55	0.13	1.95	1	1	Water
Burkholderia sp. 4NA327B6	144	7929	99.81	0.06	1.02	2	1	Water
Burkholderia sp. 4NA327C10	149	8311	99.83	0.11	1.01	1	1	Water
Burkholderia sp. 4NA327C6	123	7926	99.83	0.06	0.96	1	1	Water
Burkholderia sp. FXe9	4	8371	98.31	5.85	8.74	1	1	Soil
Burkholderia sp. SRS 25	68	7155	99.27	0.11	0.86	0	0	Soil

Supplementary Table S9. Restrictive lifestyle-associated genes (LAGs, present in ≥60% of soil free-living genomes based on bacLIFE classification) identified for the soil free-living category in *Pseudomonas paracarnis* TP30.

Level	Group	Cluster	Prokka description
Restrictive	Free living	cluster_000095	Hypothetical protein
		cluster_000562	Hypothetical protein
		cluster_000568	Hypothetical protein
		cluster_000811	Hypothetical protein
		cluster_001399	Hypothetical protein
		cluster_001442	Hypothetical protein
		cluster_001480	Hypothetical protein
		cluster_001841	Hypothetical protein
		cluster_001853	Hypothetical protein
		cluster_001927	Hypothetical protein
		cluster_002010	Hypothetical protein
		cluster_002018	Hypothetical protein
		cluster_002104	Hypothetical protein
		cluster_002538	Hypothetical protein
		cluster_002754	Hypothetical protein
		cluster_002756	Hypothetical protein
		cluster_002847	Hypothetical protein
		cluster_003105	Hypothetical protein
		cluster_003234	Hypothetical protein
		cluster_003821	Hypothetical protein
		cluster_003844	Hypothetical protein
		cluster_004052	Hypothetical protein

Level	Group	Cluster	Prokka description
		cluster_004101	Hypothetical protein
		cluster_004115	Hypothetical protein
		cluster_004332	Hypothetical protein
		cluster_004335	Hypothetical protein
		cluster_004360	Hypothetical protein
		cluster_004561	Hypothetical protein
		cluster_004606	Hypothetical protein
		cluster_004779	Hypothetical protein
		cluster_005564	Hypothetical protein
		cluster_006231	Hypothetical protein
		cluster_006630	Hypothetical protein
		cluster_006877	Hypothetical protein
		cluster_006890	Hypothetical protein
		cluster_006999	Hypothetical protein
		cluster_007078	Hypothetical protein
		cluster_007223	Hypothetical protein
		cluster_007226	Hypothetical protein
		cluster_007336	Hypothetical protein
		cluster_007406	Hypothetical protein
		cluster_007413	Hypothetical protein
		cluster_007417	Hypothetical protein
		cluster_007574	Hypothetical protein
		cluster_007580	Hypothetical protein
		cluster_007616	Hypothetical protein
		cluster_007632	Hypothetical protein
		cluster_007636	Hypothetical protein
		cluster_008104	Hypothetical protein
		cluster_012218	Hypothetical protein
		cluster_013114	Hypothetical protein
		cluster_000271	Tyrosine recombinase XerC
		cluster_000426	Nucleoid occlusion factor SlmA
		cluster_001476	putative tautomerase
		cluster_001677	Thioredoxin 1
		cluster_002444	ADP-L-glycero-D-manno-heptose-6-epimerase
		cluster_002465	Cloacin immunity protein
		cluster_002820	2,3,4,5-tetrahydropyridine-2,6-dicarboxylate N-acetyltransferase
		cluster_003223	Isochorismate pyruvate lyase

Level	Group	Cluster	Prokka description
		cluster_004313	Fimbrial subunit type 1
		cluster_006670	Phosphoenolpyruvate carboxylase
		cluster_006758	Aurachin B dehydrogenase
		cluster_006765	3-keto-5-aminohexanoate cleavage enzyme
		cluster_006848	Isobutylamine N-hydroxylase
		cluster_006856	Ferric-anguibactin-binding protein FatB
		cluster_006892	Histidine decarboxylase
		cluster_007002	Acyl-homoserine-lactone synthase
		cluster_007046	Ferric-anguibactin transport system permease protein FatC
		cluster_007097	D-inositol-3-phosphate glycosyltransferase
		cluster_007315	Isochorismate synthase EntC
		cluster_007633	Ferric-anguibactin transport system permease protein FatD
		cluster_007713	Dapdiamide A synthase
		cluster_007859	Lipid III flippase
		cluster_007896	O-mycaminosyltylonolide 6-deoxyallosyltransferase
		cluster_007968	Proline dehydrogenase 1
		cluster_008961	putative HTH-type transcriptional regulator YddM
		cluster_010757	HTH-type transcriptional activator RhaR

Supplementary Table S10. Lax lifestyle-associated genes (LAGs, present in ≥40% of genomes within a lifestyle based on bacLIFE) identified in *Burkholderia contaminans* TR100 for the soil free-living and human-opportunistic categories.

Level	Group	Cluster	Prokka description
Laxo	Free living	cluster_000482	Hypothetical protein
		cluster_001148	Hypothetical protein
		cluster_001757	Hypothetical protein
		cluster_003130	Hypothetical protein
		cluster_003551	Hypothetical protein
		cluster_004420	Hypothetical protein
		cluster_005855	Hypothetical protein
		cluster_006154	Hypothetical protein
		cluster_007256	Hypothetical protein
		cluster_008135	Hypothetical protein
		cluster_000068	Prophage integrase IntA
		cluster_000383	tRNA(fMet)-specific endonuclease VapC
		cluster_001038	Soluble epoxide hydrolase
		cluster_001490	Threonine efflux protein

Level	Group	Cluster	Prokka description
		cluster_003350	Beta-alanine degradation protein BauB
		cluster_003800	PKHD-type hydroxylase YbiX
		cluster_004958	HTH-type transcriptional regulator HdfR
		cluster_006615	Pyridoxine 4-dehydrogenase
	Human opportunistic	cluster_000057	Hypothetical protein
		cluster_000377	Hypothetical protein
		cluster_002088	Hypothetical protein
		cluster_002193	Hypothetical protein
		cluster_002301	Hypothetical protein
		cluster_002355	Hypothetical protein
		cluster_002458	Hypothetical protein
		cluster_002461	Hypothetical protein
		cluster_002893	Hypothetical protein
		cluster_002928	Hypothetical protein
		cluster_003048	Hypothetical protein
		cluster_003369	Hypothetical protein
		cluster_003690	Hypothetical protein
		cluster_003699	Hypothetical protein
		cluster_003770	Hypothetical protein
		cluster_004282	Hypothetical protein
		cluster_004335	Hypothetical protein
		cluster_004403	Hypothetical protein
		cluster_004452	Hypothetical protein
		cluster_004519	Hypothetical protein
		cluster_004621	Hypothetical protein
		cluster_004803	Hypothetical protein
		cluster_004927	Hypothetical protein
		cluster_005125	Hypothetical protein
		cluster_005892	Hypothetical protein
		cluster_005965	Hypothetical protein
		cluster_005998	Hypothetical protein
		cluster_006133	Hypothetical protein
		cluster_006140	Hypothetical protein
		cluster_006152	Hypothetical protein
		cluster_006336	Hypothetical protein
		cluster_006342	Hypothetical protein
		cluster_006357	Hypothetical protein
		cluster_006377	Hypothetical protein

Level	Group	Cluster	Prokka description
		cluster_006426	Hypothetical protein
		cluster_006490	Hypothetical protein
		cluster_007137	Hypothetical protein
		cluster_007630	Hypothetical protein
		cluster_007692	Hypothetical protein
		cluster_007707	Hypothetical protein
		cluster_007857	Hypothetical protein
		cluster_007858	Hypothetical protein
		cluster_007897	Hypothetical protein
		cluster_008070	Hypothetical protein
		cluster_008293	Hypothetical protein
		cluster_008351	Hypothetical protein
		cluster_008584	Hypothetical protein
		cluster_008672	Hypothetical protein
		cluster_008733	Hypothetical protein
		cluster_008775	Hypothetical protein
		cluster_008792	Hypothetical protein
		cluster_009028	Hypothetical protein
		cluster_009138	Hypothetical protein
		cluster_009234	Hypothetical protein
		cluster_000052	Tn3 family transposase ISBusp1
		cluster_000107	Tyrosine recombinase XerC
		cluster_000113	Putative nuclease YhcG
		cluster_000775	IS200/IS605 family transposase ISAba30
		cluster_001053	Sorbitol dehydrogenase
		cluster_001183	2,6-dihydropseudooxynicotine hydrolase
		cluster_002486	Putative non-heme bromoperoxidase BpoC
		cluster_002778	Kynurenine formamidase
		cluster_002975	Nitrogen regulatory protein P-II
		cluster_003893	Copper resistance protein A
		cluster_003998	mRNA interferase toxin RelE
		cluster_004061	Thiol-disulfide oxidoreductase ResA
		cluster_004303	Plasmid replication initiator protein TrfA
		cluster_004402	putative MFS-type transporter YhhS
		cluster_005435	Pyrroline-5-carboxylate reductase
		cluster_006256	Membrane-bound lytic murein transglycosylase F
		cluster_006409	Sodium-dependent dicarboxylate transporter SdcS
		cluster_006474	Adenylyl-sulfate kinase

Level	Group	Cluster	Prokka description
		cluster_007346	Cell division coordinator CpoB
		cluster_008355	Lactate utilization protein A
		cluster_008531	Renalase
		cluster_008969	Lactate utilization protein C

REFERENCES

1. Chen YS, Shieh WJ, Goldsmith CS, Metcalfe MG, Greer PW, Zaki SR, Chang HH, Chan H, Chen YL. 2014. Alteration of the phenotypic and pathogenic Patterns of Burkholderia pseudomallei that persist in a soil environment. Am J Trop Med Hyg 90:469–479.
2. Nagarajan D, Aristya GR, Lin Y-J, Chang J-J, Yen H-W, Chang J-S. 2021. Microbial cell factories for the production of polyhydroxyalkanoates. Essays Biochem 65:337–353.
3. Wendy YD, Fauziah MN, Baidurah Y., Tong WY, Lee CK. 2022. Production and characterization of polyhydroxybutyrate (PHB) BY Burkholderia cepacia BPT1213 using waste glycerol as carbon source. Biocatal Agric Biotechnol 41:102310.
4. Chang J, Yan Z, Dong J, Wu X, Meng Z, Shi Y, Chen J. 2022. Mechanisms controlling the transformation of and resistance to mercury(II) for a plant-associated Pseudomonas sp. strain, AN-B15. J Hazard Mater 425:127948.
5. Cardona GI, Escobar MC, Acosta-González A, Marín P, Marqués S. 2022. Highly mercury-resistant strains from different Colombian Amazon ecosystems affected by artisanal gold mining activities. Appl Microbiol Biotechnol 106:2775–2793.
6. Bochkareva OO, Moroz E V., Davydov II, Gelfand MS. 2018. Genome rearrangements and selection in multi-chromosome bacteria Burkholderia spp. BMC Genomics 19:1–17.
7. Seemann T. 2014. Prokka: Rapid prokaryotic genome annotation. Bioinformatics 30:2068–2069.
8. Albright S, Louca S. 2023. Trait biases in microbial reference genomes. Sci Data 10:1–17.
9. Xu H, Qin S, Lan Y, Liu M, Cao X, Qiao D, Cao Y, Cao Y. 2017. Comparative genomic analysis of Paenibacillus sp. SSG-1 and its closely related strains reveals the effect of glycometabolism on environmental adaptation. Sci Rep 7:1–11.
10. Zhou J, Du B, Shang L, Wang Z, Cui H, Fan X, Zhou J. 2020. Mercury fluxes, budgets, and pools in forest ecosystems of China: A review. Crit Rev Environ Sci Technol 50:1411–1450.
11. Lan Y, Liu M, Song Y, Cao Y, Li F, Luo D, Qiao D. 2023. Distribution, characterization, and

evolution of heavy metal resistance genes and Tn7-like associated heavy metal resistance Gene Island of Burkholderia. Front Microbiol 14:1–16.

12. Stanton CR, Petrovski S, Batinovic S. 2024. Isolation of a PRD1-like phage uncovers the carriage of three putative conjugative plasmids in clinical Burkholderia contaminans. Res Microbiol 175:104202.

13. Lobb B, Tremblay BJM, Moreno-Hagelsieb G, Doxey AC. 2020. An assessment of genome annotation coverage across the bacterial tree of life. Microb Genomics 6:e000341.
